# Strategies for Poly(3-hydroxybutyrate) Production Using a Cold-Shock Promoter in *Escherichia coli*

**DOI:** 10.3389/fbioe.2021.666036

**Published:** 2021-06-03

**Authors:** Thanawat Boontip, Rungaroon Waditee-Sirisattha, Kohsuke Honda, Suchada Chanprateep Napathorn

**Affiliations:** ^1^Department of Microbiology, Faculty of Science, Chulalongkorn University, Bangkok, Thailand; ^2^International Center for Biotechnology, Osaka University, Suita, Japan

**Keywords:** polyhydroxybutyrate, pCold, cold shock, *Cupriavidus necator*, *E. coli* – *Escherichia coli*, cspA gene

## Abstract

The present study attempted to increase poly(3-hydroxybutyrate) (PHB) production by improving expression of PHB biosynthesis operon derived from *Cupriavidus necator* strain A-04 using various types of promoters. The intact PHB biosynthesis operon of *C. necator* A-04, an alkaline tolerant strain isolated in Thailand with a high degree of 16S rRNA sequence similarity with *C. necator* H16, was subcloned into pGEX-6P-1, pColdI, pColdTF, pBAD/Thio-TOPO, and pUC19 (native promoter) and transformed into *Escherichia coli* JM109. While the *phaC*_A–04_ gene was insoluble in most expression systems tested, it became soluble when it was expressed as a fusion protein with trigger factor (TF), a ribosome associated bacterial chaperone, under the control of a cold shock promoter. Careful optimization indicates that the cold-shock cspA promoter enhanced phaC_A–04_ protein expression and the chaperone function of TF play critical roles in increasing soluble phaC_A–04_ protein. Induction strategies and parameters in flask experiments were optimized to obtain high expression of soluble PhaC_A–04_ protein with high Y_P/S_ and PHB productivity. Soluble phaC_A–04_ was purified through immobilized metal affinity chromatography (IMAC). The results demonstrated that the soluble phaC_A–04_ from pColdTF-*phaCAB*_A–04_ was expressed at a level of as high as 47.4 ± 2.4% of total protein and pColdTF-*phaCAB*_A–04_ enhanced soluble protein formation to approximately 3.09−4.1 times higher than that from pColdI-*phaCAB*_A–04_ by both conventional method and short induction method developed in this study. Cultivation in a 5-L fermenter led to PHB production of 89.8 ± 2.3% PHB content, a Y_P/S_ value of 0.38 g PHB/g glucose and a productivity of 0.43 g PHB/(L.h) using pColdTF-*phaCAB*_A–04_. The PHB film exhibited high optical transparency and possessed M_w_ 5.79 × 10^5^ Da, M_n_ 1.86 × 10^5^ Da, and PDI 3.11 with normal melting temperature and mechanical properties.

## Introduction

The global environmental concern regarding microplastics in the marine environment as contaminants with significant impacts on animal and human health has led to a call for national and international policies from more than 60 countries to ban or place a levy on single-use plastics (Steensgaard et al., 2017; Xanthos and Walker, 2017; Schnurr et al., 2018; Prata et al., 2019). Renowned global companies have also integrated regulations and policies to ban single-use plastics into their green marketing and corporate social responsibility policies. Bioplastics are becoming a popular alternative to single-use plastics to reduce the amount of microplastic waste. Recently, the role of compostable plastics within the circular economy has been highlighted. To establish a truly circular economy, the EU focuses on the contribution of biodegradable and compostable plastics to help the EU to meet its organic waste recovery targets by the end of 2023. The market for bioplastics is growing and the demand for bioplastics is rising. European Bioplastics reported that the global bioplastic production capacity will increase by 36 percent from 2.1 million tons in 2020 to approximately 2.8 million tons in 2025 (Bie, 2020). Among the various types of bioplastics, polyhydroxyalkanoates (PHAs) are an important biodegradable polymer family, as they are one hundred percent biobased and fully biodegradable in all environments, especially marine (ASTM 7081) and fresh water environments (Gross and Kalra, 2002; Volova et al., 2007).

To obtain both the environmental and economic benefits of PHAs over synthetic plastics and other bioplastics, microorganisms that exhibit efficient PHA production from inexpensive and renewable carbon sources are urgently required to develop a low-cost approach. Microbial cells typically accumulate PHA at approximately 30–50% of the cell dry mass (CDM). The best known industrial PHA producer, *Cupriavidus necator* H16 (formerly known as *Alcaligenes eutrophus*, *Ralstonia eutropha*, and *Wautersia eutropha*), is capable of accumulating polyhydroxybytyrate (PHB) at over 80% of the CDM. PHA accumulation is tightly regulated by imbalanced growth conditions with excess carbon but limited nitrogen (Kawaguchi and Doi, 1992). One of the major limitations in the production of PHAs in wild-type strains has been intracellular polymer degradation caused by endogenous PHA depolymerases, which is different from the behavior of exogenous PHA depolymerases (Gebauer and Jendrossek, 2006). Therefore, intracellular PHAs are often spontaneously degraded during cultivation when the bacteria require carbon, resulting in low PHA content and a wide range of molecular weight distributions in wild-type strains. Thus, many recombinant strains have been developed by metabolic engineering to obtain a high yield of PHB and a molecular weight that is high enough for polymer processing (Liu et al., 1998; Ahn et al., 2000; Kahar et al., 2005; Taguchi et al., 2005; Agus et al., 2006b; Hiroe et al., 2012). Ordinarily, the PHB biosynthesis pathway begins with acetyl-CoA and requires three major enzymes, namely, 3-ketothiolase (*phaA*), NADPH-dependent acetoacetyl-CoA reductase (*phaB*), and PHA synthase (*phaC*), and these three genes are sufficient for the production of PHB in non-PHA-producing bacteria at more than 90% of the CDM when heterologously expressed in *Escherichia coli* (Lee et al., 1994). It has been reported that PhaC plays a key role in obtaining the polymeric form, resulting in a high level and high molecular weight of PHB (Kahar et al., 2005; Agus et al., 2006b).

To date, PHA synthases have been categorized into four major classes based on their sequence, substrate specificity, and subunit composition (Rehm, 2003; Tsuge, 2016). It was reported that PhaC derived from *C. necator* H16 (PhaC_H16_) is a Class I PhaC and is one of the most widely studied PHA synthases. It has a molecular weight of approximately 64 kDa (589 amino acids) and is located as the first gene in the PHA biosynthetic phaCAB operon, followed by PhaA and PhaB (Schubert et al., 1988; Peoples and Sinskey, 1989). It was demonstrated that the weight-average molecular weight (M_w_) of PHB synthesized by wild-type bacteria is generally in the range of 0.1–2.0 × 10^6^ Da. When recombinant PhaC_H16_ was overexpressed in *E. coli*, most of the protein formed insoluble inclusion bodies due to its low aqueous solubility (Gerngross et al., 1994; Gerngross and Martin, 1995; Zhang et al., 2000; Yuan et al., 2001). To feasibly achieve industrial-scale production, PhaC would need to be produced in large quantities and its solubility would need to be improved (Thomson et al., 2013). There have been many reports that have attempted to resolve the problem mentioned above, including by modulating the concentration of the PhaC protein by varying the chemical inducer quantities (Agus et al., 2006a); expressing the protein at a reduced temperature (30°C) (Thomson et al., 2013); fusing the PhaC protein with a glutathione S-transferase (GST) tag, which is a hydrophilic tag, to improve its solubility (Harada et al., 2019); and coexpressing the protein with chaperones to obtain high total quantities of enzyme and a larger proportion in the soluble fraction than obtained without chaperones. In this study, we reported the use of pCold (cspA promoter) to improve PhaC expression as well as its combination with trigger factor (TF) chaperone and compared with the promoters mentioned above.

In a previous study, we reported the generation of the *C. necator* strain A-04, possessing 99.78% 16S RNA sequence similarity with *C. necator* H16 but differing in PHA production ability (Chanprateep et al., 2008). Designed using the gene walk technique, the PHA biosynthesis operon of *C. necator* strain A-04 consisted of three genes, encoding acetyl-CoA acetyltransferase (*phaA*_A–04_, 1182 bp, 40.6 kDa, accession no. FJ897461), acetoacetyl-CoA reductase (*phaB*_A–04_, 741 bp, 26.4 kDa, accession no. FJ897462) and PHB synthase (*phaC*_A–04_, 1770 bp, 64.3 kDa, accession no. FJ897463). Sequence analysis of the *phaA*_A–04_, *phaB*_A–04_, and *phaC*_A–04_ genes revealed that *phaC*_A–04_ was 99% similar to *phaC*_H16_ from *C. necator* H16. The difference was in the amino acid residue situated at position 122, which in *phaC*_A–04_ was proline but in *C. necator* H16 was leucine. The total amino acid sequences of *phaA*_A–04_ and *phaB*_A–04_ were 100% matched with those of *C. necator* H16 (Napathorn et al., 2021). Notably, *C. necator* strain A-04 prefers fructose over glucose as a carbon source, accumulating PHB at 78% of the CDM under a C/N ratio of 200, whereas it could incorporate a high mole fraction of monomeric 4-hydroxybutyrate monomeric into the poly(3-hydroxybutyrate-*co*-4-hydroxybutyrate) [P(3HB-*co*-4HB)] copolymer under a C/N ratio of 20 (Chanprateep et al., 2010), as well as the poly(3-hydroxybutyrate-*co*-3-hydroxyvaterate-*co-*4-hydroxybutyrate) [P(3HB-*co-*3HV-*co-*4HB)] terpolymer (Chanprateep and Kulpreecha, 2006). In the prior study, the intact *phaCAB*_A–04_ operon was cloned into the arabinose-inducible araBAD promoter and transformed into *E. coli* strains Top 10, JM109 and XL-1 blue. The results showed that optimal conditions obtained from shaken flask experiments yielded 6.1 ± 1.1 g/L cell dry mass (CDM), a PHB content of 93.3 ± 0.9% (w/w) and a productivity of 0.24 g/(L⋅h). Finally, fed-batch cultivations by pH-stat control in a 5-L fermenter of *E. coli* strains XL1-Blue harboring pBAD/Thio-TOPO-*phaCAB*_A–04_, leading the PHB production of 29.0 ± 1.1 g/L with 60.2 ± 2.3% PHB content in the cell dry mass (CDM) of 53.1 ± 1.0 g/L, a Y_P/S_ value of 0.21 g PHB/g glucose and a productivity of 0.4 g PHB/(L⋅h) in LB medium.

**FIGURE 1:**
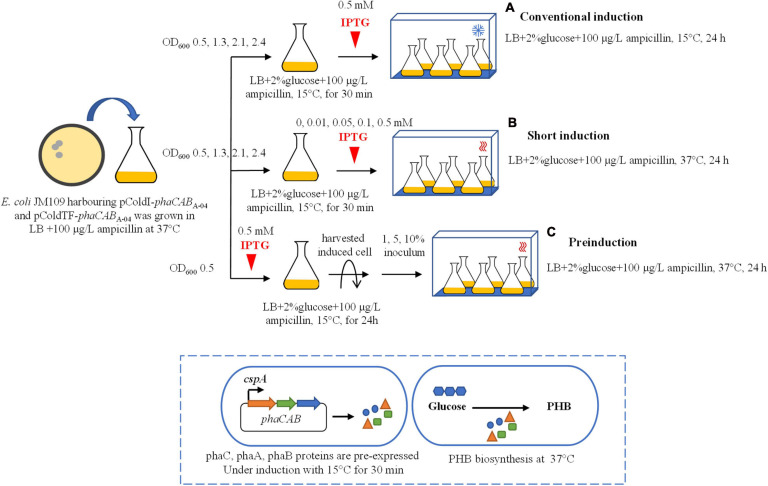
Schematic of three different induction methods for heterologous expression of the *phaCAB*_A–04_ biosynthesis operon in *E. coli* JM109 (pColdI-*phaCAB*_A–04_) and *E. coli* JM109 (pColdTF-*phaCAB*_A–04_). **(A)** Conventional induction method: the culture was incubated at 37°C and 200 rpm until the OD_600_ reached 0.5, 1.3, 2.1, and 2.4. Then, the cultivation temperature was decreased from 37 to 15°C for 30 min, and the expression of the *phaCAB*_A–04_ operon was induced by the addition of 0.5 mM IPTG. The cultivation temperature was further maintained at 15°C for 24 h. **(B)** Short-induction method: the culture was incubated at 37°C and 200 rpm until the OD_600_ reached 0.5, 1.3, 2.1, and 2.4. Then, the temperatures were varied at 15, 25, 30, and 37°C for 30 min. Next, the expression of the *phaCAB*_A–04_ operon was induced by adding various concentrations (0.01, 0.05, 0.1, 0.5, and 1.0 mM) of IPTG, and the cultivation temperature was maintained at 37°C for 24 h. **(C)** Preinduction method: the culture was incubated at 37°C and 200 rpm until the OD_600_ reached 0.5. Next, 0.5 mM IPTG was added into the culture when the temperature was decreased from 37°C to 15°C for 24 h. Then, the induced cells were harvested by centrifugation, the medium was discarded, and the cells were resuspended in an equal volume of fresh LB medium. Finally, the induced cells at 1, 5, or 10% (v/v) were transferred into fresh LB medium supplemented with 100 μg/L ampicillin and 20 g/L glucose and incubated at 37°C and 200 rpm for 24 h.

Thus, the objective of this work was to express *phaCAB*_A–04_ genes from the isolated *C. necator* strain A-04 in pColdI (cspA promoter, cold- and IPTG-inducible vector, N-terminal 6His-fusion protein) and pColdTF (cspA promoter, cold- and IPTG-inducible vector, trigger factor (TF) chaperone, N-terminal 6His-fusion protein). The reason for choosing pColdI and pColdTF is that there have been few reports on utilizing cold inducible promoter for PHB production. Therefore, the obtained results will prove that if phaC solubility is enhanced, this outcome would finally result in enhancing PHB production. It has been well known that CspA was originally found as the major cold-shock protein in *E. coli*, consisting of 70-amino-acid residues. CspA forms a β-barrel structure with five anti-parallel β-strands and functions as an RNA chaperone. Its transient induction upon cold shock is regulated at the level of transcription, mRNA stability and translation (Yamanaka et al., 1998). Cold shock proteins are not only produced during cold stress, but in *E. coli*, CspA also forms 1% of all soluble proteins at the early exponential growth phase at 37°C, suggesting that CspA also functions at optimal growth temperature (Brandi et al., 1999). Furthermore, insolubility of protein overexpressed in *E. coli* is a common problem. Therefore, co-expression with solubility enhancers was utilized to resolve these issues, such as GST and TF. TF is a prokaryotic ribosome associated chaperone (MW 48 kDa), which facilitates co-translational protein folding, thus, reducing the chances of forming misfolded and insoluble proteins (Patzelt et al., 2002; Maier et al., 2005). As a native product from prokaryotes, TF is highly expressed in *E. coli*, which allowed for high yield of recombinant proteins (Baneyx, 1999). Another benefit of using the pCold system is that the induction was carried out at cold temperatures, which has been shown to significantly improve protein folding by decreasing the rate of transcription and translation, thus providing more time for the protein to be folded (Agashe et al., 2004; Baneyx and Mujacic, 2004). Compared with previous reports, the PHA biosynthesis operon of *C. necator* strain A-04 was also cloned into pGEX-6P-1 [tac promoter, isopropyl-β-D-thiogalactopyranoside (IPTG)-inducible vector, N-terminal GST fusion protein], pBAD/Thio-TOPO (araBAD promoter, arabinose-inducible vector, N-terminal thioredoxin fusion protein and C-terminal 6His-fusion protein) (Napathorn et al., 2021) and pUC19 (control strain, *phaCAB*_A–04_ biosynthesis genes with native promoter of *phaC*_A–04_) and transformed into *E. coli* JM109 (Table 1). Next, to optimize phaC_A–04_ overexpression in shake flask cultivation, three induction methods were tested and compared with conventional induction method (Figure 1, details are described in the section “Materials and Methods”). The effect of phaC_A–04_ overexpression on PHB production in recombinant *E. coli* with respect to cell growth, glucose consumption, PHB production, and kinetic parameters in conditions ranging from flask culture to a 5-L fermenter. PhaC_A–04_ was purified, quantified, carefully compared versus pColdI-*phaCAB*_A–04_ and pColdTF-*phaCAB*_A–04_, and also between short induction and conventional induction methods. Furthermore, to examine the effect of cspA promoter and TF chaperone on the polymer properties, the produced PHB was subjected to molecular weight determination, thermal analysis and mechanical property measurement.

**TABLE 1:** Bacterial strains and plasmids used in this study.

Strains/plasmids	Relevant description	Reference/source
Strain *Cupriavidus necator* strain A-04	Wild Type	
*Escherichia coli* JM109	F′*traD36 proA+B+ lacI^q^(lacZ)ΔM15/Δ(lac-proAB) glnV44 e14- gyrA96 recA1 relA1 endA1 thi hsdR17*	Promega Corporation, Madison, WI, United States
**Plasmid**		
pUC19	Amp^r^	Thermo Scientific, MA, United States
pColdI	Amp^r^, lacI, cold-shock cspA promoter	Takara Bio Inc., Shiga, Japan
pColdIF	Amp^r^, lacI, cold-shock cspA promoter and trigger factor	Takara Bio Inc., Shiga, Japan
pGEX-6P-1	Amp^r^, lacI, tac promoter and glutathione S-transferase (GST)	Novagen, WI, United States
pBAD/Thio-TOPO	Amp^r^, araBAD promoter and thioredoxin	Invitrogen, CA, United States
pUC19-nativeP- *phaCAB*_A–04_	pUC19 derivative, carrying *phaCAB* with native promoter from *C. necator* strain A-04	This study
pColdI-*phaCAB*_A–04_	pColdI derivative, carrying N-terminal 6His-fused *phaCAB* from *C. necator* strain A-04	This study
pColdTF-*phaCAB*_A–04_	pColdTF derivative, carrying N-terminal 6His-fused *phaCAB* from *C. necator* strain A-04	This study
pGEX-6P-1- *phaCAB*_A–04_	pGEX-6P-1 derivative, carrying N-terminal GST and 6His-fused *phaCAB* from *C. necator* strain A-04	This study
pBAD/Thio-TOPO- *phaCAB*_A–04_	pBAD/Thio-TOPO^®^ derivative, carrying C-terminal 6HIS- and N-terminal thioredoxin fused *phaCAB* from *C. necator* strain A-04	Napathorn et al., 2021
**Primer**		
pCold-F	5′-ATGGATCCCTCGAGATGGCGA CCGGCAAAG-3′	This study
pCold-R	5′-GTGAATTCAAGCTTTCAGCCCATAT GCAGGCC-3′	This study
pGEX-F	5′-GGCCCCTGGGATCCCCGGAAATG GCGACCGGCAA-3′	This study
pGEX-R	5′- GCACTCGACTCGAGTCAGCCCAT ATGCAGG-3′	This study
nativeP-*phaCAB*_A–04_-F	5′-TGGTCCCTGA CTGGC-3′	This study
nativeP-*phaCAB*_A–04_-R	5′-CGTCGACGACC TTGAAT-3′	This study

## Materials and Methods

### Strains and Plasmids

The *E. coli* strains and plasmids used in this study are listed in Table 1. The PHB-producing *C. necator* strain A-04 (Chanprateep et al., 2008) was used to isolate the *phaCAB*_A–04_ gene operon. All bacterial strains were grown at 37°C in Luria-Bertani (LB) medium supplemented with 100 μg/L ampicillin. The LB medium contained (per liter) 10 g of tryptone (Himedia, Mumbai, India), 5 g of yeast extract (Himedia, Mumbai, India) and 10 g of NaCl (Merck KGaA, Darmstadt, Germany). Stock cultures were maintained at –80°C in a 15% glycerol solution. The experiments were performed in a biosafety level 1 laboratory and by researchers and investigators who had undergone biosafety training.

### Construction of Recombinant Plasmids

The *phaCAB*_A–04_ operon PHB biosynthetic genes from *C. necator* A-04 were PCR-amplified using the following pair of primers: forward primer 5′-ATGGATCC CTCGAGATGGCGACCGGCAAAG-3′ (the XhoI site is underlined) and reverse primer 5′-GTGAATTCAAGCTT TCAGCCCATATGCAGGCC-3′ (the HindIII site is underlined). Primers were designed based on accession numbers FJ897463, FJ897461, and FJ897462. The blunted PCR product was purified and subcloned into pBluescript SK- (Stratagene, La Jolla, CA, United States) linearized by SmaI. The recombinant plasmid digested with XhoI and HindIII was cloned into cold-shock-inducible pColdI and pColdTF vectors (Takara Bio Inc., Shiga, Japan) at the XhoI and HindIII restriction sites, yielding pColdI-phaCAB_A–04_ and pColdTF-phaCAB_A–04_, respectively. For the plasmid pGEX-6P-1-phaCAB_A–04_, the phaCAB_A–04_ operon was amplified by the primers pGEX-F and pGEX-R (Table 1). The 3,885-bp DNA fragment was digested by BamHI and XhoI and cloned into BamHI-XhoI-digested pGEX-6P-1 to obtain pGEX-6P-1-*phaCAB*_A–04_. To construct pUC19-nativeP-*phaCAB*_A–04_, the primers nativeP-*phaCAB*_A–04_-F and nativeP-*phaCAB*_A–04_-R were used to amplify the *phaCAB*_A–04_ operon, including its native promoter. The blunted PCR product was purified and cloned into SmaI-linearized pUC19 (Thermo Fisher Scientific, Inc., Waltham, MA, United States), yielding pUC19-nativeP-*phaCAB*_A–04_. PCRs were performed using Q5^®^ High-Fidelity DNA Polymerase (New England Biolabs, Ipswich, MA, United States). *E. coli* JM109 was used as a host for cloning and PHB production. The accuracy of the constructed plasmid was verified by the corresponding restriction enzyme and sequencing.

### Optimization of Culture Conditions for PHB Production in Shaken Flask Cultivation

Expression vectors named pColdI-*phaCAB*_A–04_ and pColdTF-*phaCAB*_A–04_ with the entire phaCAB_A–04_ operon were transformed into *E. coli* JM109 by the heat shock method (Sambrook and Russell, 2001). Shake flask experiments were performed in 250-mL Erlenmeyer flasks containing 50 mL of medium. *E. coli* JM109 cells transformed with pColdI-*phaCAB*_A–04_ or pColdTF-*phaCAB*_A–04_ were grown in LB medium containing ampicillin (100 μg/mL) on a rotary incubator shaker (Innova 4300, New Brunswick Scientific Co., Inc., Edison, NJ, United States) at 37°C and 200 rpm for 24 h. The overnight seed culture was inoculated into fresh LB medium (5% v/v inoculum) containing 100 μg/L ampicillin and 20 g/L glucose prior to induction with temperature and IPTG using three separate induction methods (Figure 1).

For the synthesis of PHB using the conventional induction method, the procedure was performed according to the user manual (Takara Bio Inc., Otsu, Shiga, Japan). The culture was incubated at 37°C and 200 rpm until the optical density at 600 nm (OD_600_) reached 0.5, 1.3, 2.1, and 2.4. Next, the cultivation temperature was reduced from 37°C to 15°C for 30 min. The expression of the *phaCAB* operon was induced by the addition of 0.5 mM IPTG, and cultivation was continued at 15°C for an additional 24 h.

For the synthesis of PHB using the short-induction method developed in this study, the culture was incubated at 37°C and 200 rpm until the OD_600_ reached 0.5, 1.3, 2.1, and 2.4. Then, the temperatures were varied at 15, 25, 30, and 37°C for 30 min. Next, the expression of the *phaCAB* operon was induced by adding various concentrations (0.01, 0.05, 0.1, 0.5, and 1.0 mM) of IPTG, and the cultivation was maintained at 37°C for 24 h.

For the synthesis of PHB using the preinduction method developed in this study, the culture was incubated at 37°C and 200 rpm until the OD_600_ reached 0.5. Then, 0.5 mM IPTG was added to the culture and the temperature was reduced from 37°C to 15°C for 24 h. The induced cells were harvested by centrifugation, the medium was discarded, and the cells were resuspended in an equal volume of fresh LB medium. Then, the induced cells at 1, 5, or 10% (v/v) were transferred into fresh LB medium supplemented with 100 μg/L ampicillin and 20 g/L glucose and incubated at 37°C and 200 rpm for 24 h.

For comparison of the effect of phaC expression on PHB production under various types of promoters, fusion proteins and chaperones, shake flask experiments were performed in 250-mL Erlenmeyer flasks containing 50 mL of LB medium containing ampicillin (100 μg/mL) on a rotary incubator shaker at 37°C and 200 rpm for 24 h. For PHB production, overnight cultures in LB medium (1 mL) were transferred into fresh LB medium supplemented with glucose (20 g/L) and ampicillin (100 μg/mL). Recombinant *E. coli* JM109 (pColdI-*phaCAB*_A–04_) and *E. coli* JM109 (pColdTF-*phaCAB*_A–04_) were induced to produce PHB using the conventional induction method and short-induction method. The effect of GST (the hydrophilic fusion protein) and the tac promoter on PHB production was investigated using *E. coli* JM109 (pGEX-6P-1-*phaCAB*_A–04_), which was induced by the addition of IPTG (0.5 mM). The effect of the araBAD promoter and N-terminal thioredoxin fusion protein together with the C-terminal 6His-fusion protein on PhaC and PHB production was examined by inducing *E. coli* JM109 (pBAD/Thio-TOPO-*phaCAB*_A–04_) with arabinose (1% w/v). *E. coli* JM109 (pUC19-nativeP-*phaCAB*_A–04_), which exhibits expression from native promoter without addition of IPTG, was used as a control strain. All of these comparison experiments were performed at 15 or 37°C for 48 h. The crude glycerol used in this study was obtained from biodiesel industries belong to Bangchak Corporation Public Company Limited, a petroleum and energy conglomerate in Thailand. The content of glycerol was 80% w/v.

### Conditions for PHB Production in a 5-L Fermentor

A preculture was prepared in 500-mL Erlenmeyer flasks containing 100 mL of LB medium and grown on a rotary shaker at 37°C at 200 rpm for 24 h. The preculture was inoculated into a 5-L bioreactor (MDL500, B.E. Marubishi Co., Ltd., Tokyo, Japan) containing 2 L of LB medium supplemented with 100 μg/L ampicillin and 20 g/L glucose at an inoculation volume of 5% (v/v). The agitation speed and the air flow rate were 500 rpm and 1 mL/min, respectively. After an OD_600_ of 0.5 was obtained, the cultivation temperature was reduced from 37 to 15°C for 30 min. Next, IPTG was added to the culture at a final concentration of 0.5 mM. After IPTG addition, the cultivation temperature was shifted from 15 to 37°C and maintained at 37°C for 48 h. Culture samples were collected at 6 h intervals for 48 h.

### Analytical Methods

Cell growth was monitored by the CDM, which was determined by filtering 5 mL of the culture broth through preweighed cellulose nitrate membrane filters (pore size = 0.22 μm; Sartorius, Goettingen, Germany). The filters were dried at 80°C for 2 days and stored in desiccators. The net biomass was defined as the residual cell mass (RCM), which was calculated by subtracting the amount of PHB from the CDM. The PHB in dried cells was methyl-esterified using a mixture of chloroform and 3% (v/v) methanol-sulfuric acid (1:1 v/v) (Braunegg et al., 1978). The resulting monomeric methyl esters were quantified by a gas chromatograph (model CP3800, Varian Inc., Walnut Creek, CA, United States) using a Carbowax-PEG capillary column (0.25-μm df, 0.25-mm ID, 60-m length, Varian Inc.). The internal standard was benzoic acid, and the external standard was PHB (Sigma-Aldrich Corp.). The total reducing sugar concentration was determined using a 3,5-dinitrosalicylic acid (DNS) assay (Miller, 1959). Glycerol and acetate concentrations in culture medium were analyzed using an HPLC system (1200 Infinity series, Agilent technologies, United States) equipped with 1260 RID (Agilent Technologies, United States) and X-bridge-BEH amide column (4.6 × 250 nm × 5 μm) (Water, United States), with an isocratic mobile phase of acetonitrile: water (70:30, v/v) at a flow rate 1.0 mL/min and 30°C (Dharmadi et al., 2006; Simonzadeh and Ronsen, 2012).

### Sodium Dodecyl Sulfate-Polyacrylamide Gel Electrophoresis (SDS-PAGE) and Western Blot Analysis

Recombinant *E. coli* cells were cultured with and without induction. Cells were collected by centrifugation at 17,000 × *g* and 4°C for 30 min. Cell pellets were resuspended in 100 mM Tris-HCl (pH 8.0) and normalized to an OD_600_ of 2.0. Total proteins were extracted from cells by using a sonicator (Sonics Vibra Cell VCX 130, Sonics & Materials, Inc., Newtown, CT, United States). The lysis mixture was then centrifuged at 17,000 × *g* at 4°C for 30 min. The protein concentration in the supernatant (soluble protein) was estimated by the Bradford method using a Bio-Rad protein assay kit (Bio-Rad Laboratories Inc., Hercules, CA, United States), and bovine serum albumin was used as a standard. Thirty micrograms of total protein from each sample was subjected to sodium dodecyl sulfate-polyacrylamide gel electrophoresis (SDS-PAGE) using 10% polyacrylamide gels under reducing conditions and electrophoresed at 80 V for 10 min followed by 140 V for 60 min. For the western blot analysis, the protein from SDS-PAGE was then transferred to a polyvinylidene difluoride (PVDF) membrane using a semi-dry blotting system (Trans-Blot SD Cell, Bio-Rad Laboratories Inc, Hercules, CA, United States) at 150 mA for 40 min. The 6His tag was detected by a mouse anti-His antibody (Aviva Systems Biology Corp., San Diego, CA, United States) and an HRP-conjugated goat anti-mouse IgG as the primary and secondary antibodies, respectively. Color development was performed using a Mouse IgG DAB Chromogenic Reagent Kit (Boster Biological Technology, Pleasanton CA, United States) according to the manufacturer’s instructions.

### Protein Purification by Immobilized Metal Affinity Chromatography (IMAC)

The cell pellet (from 50 mL culture) was resuspended in 1 ml of lysis-equilibration-wash buffer (1X LEW buffer, 50 mM NaH_2_PO_4_, 300 mM NaCl and pH 8.0). Lysozyme (USB Corporation, OH, United States) and Benzonase^®^ endonuclease (Novagen Inc., WI, United States) were added to concentrations of 0.2 mg/mL and 20 U/mL, respectively. The cells were ruptured by ultrasonic homogenizer (Vibra-Cell^TM^ Ultrasonic Liquid Processors VCX 130, Sonics & Materials, Inc., CT, United States). The amplitude was set to 40% (pulse interval at 30/15 s for 5 min). The lysate was clarified by centrifugation at 16, 100 × *g* for 20 min and the supernatant was collected. Protino@^®^ Ni-IDA 1000 His-Tag Protein purification columns (Macherey-Nagel GmbH & Co. KG, Düren, Germany) were pre-equilibrated with four bed volumes of 1X LEW buffer and allowed to drain by gravity. The cleared supernatant with 2 mg of total protein was loaded onto a pre-equilibrated column and washed with four bed volumes of 1X LEW buffer containing 20 mM imidazole. Finally, the polyhistidine-tagged protein was eluted with elution buffer (50 mM NaH_2_PO_4_, 300 mM NaCl, 250 mM imidazole and pH 8.0). Each fraction was analyzed by running 10 μL of eluate on SDS-PAGE and quantified by Bradford protein assay. To ensure accurate quantitation of yields, the lysate flow-through was collected for detection of unbound product by SDS-PAGE analysis.

### Analysis of Polymer Molecular Weight

The molecular weight was determined by Gel Permeation Chromatography (GPC; Shimadzu 10A GPC system, Shimadzu Co., Ltd., Kyoto, Japan) with a 10A refractive index detector and two Shodex columns (a GPC K-806M column (8.0 mm ID × 300 mm L, Showa Denko K.K., Tokyo, Japan) belong to Associate Professor Takeharu Tsuge’s laboratory at Department of Materials Science and Engineering, School of Materials and Chemical Technology, Tokyo Institute of Technology, Yokohama, Japan. Polymer was dissolved in 0.1% (w/v) chloroform and filtered through a 0.45 μm low protein binding Durapore^®^ (PVDF) membrane filter (Millex^®^-HV, Merck Millipore Ltd., Tullagreen, Carrigtwohill Co., Cork, Ireland). The temperature was 40°C and the flow rate was 0.8 mL/min. A standard curve was determined for polystyrene with low polydispersity in the same conditions for the molecular weight 1.26 × 10^3^, 3.39 × 10^3^, 1.30 × 10^4^, 5.22 × 10^4^, 2.19 × 10^5^, 7.29 × 10^5^, 2.33 × 10^6^, and 7.45 × 10^6^. The weight-average molecular weight (M_w_) and the number-average molecular weight (M_n_) were determined by gel permeation chromatography (GPC) and the polydispersity index (PDI) was calculated as the ratio $\frac{M_{\text{w}}}{M_{\text{n}}}$.

### Preparation of PHB Films

PHB films were prepared according to the ASTM: D882-91 protocol. The PHB films were prepared from chloroform solutions of the polyesters using conventional solvent-casting techniques and a glass tray [Pyrex, Corning Incorporated, NY, United States) as the casting surface (modified from Yoshie et al. (1995)]. The thickness of the thin polyester films was regulated by controlling the concentration of the polymer in chloroform (1% w/v) and the volume of the polymer solution. The thickness of the PHB films was 0.05 mm, which was confirmed using a caliper (Model 500-175: CD-12C, Mitutoyo Corporation, Kawasaki-shi, Kanagawa, Japan). A film samples were aged for 1 month in desiccator at ambient temperature to allow them to reach crystallization equilibrium.

### Analysis of the Mechanical Properties of PHB Films

The mechanical tests were conducted at the Scientific and Technological Research Equipment Center, Chulalongkorn University, using a universal testing machine (H10KM, Wuhan Huatian Electric Power Automation Co., Ltd., Wuhan, China) with a crosshead speed of 10 mm/min. The variables measured included the elongation at the break point (%), the stress at maximal load (MPa), and the Young’s modulus (MPa). The data represent the mean values for ten samples tested under the same conditions.

### Thermal Analysis by Differential Scanning Calorimetry (DSC) of PHB Films

A 10-mg sample of PHB was encapsulated in an aluminum sample vessel and placed in the sample holding chamber of the DSC apparatus (DSC7, PerkinElmer, Inc., Waltham, MA, United States). STARe software (version SW 10.00; Mettler-Toledo International Inc., Columbus, OH, United States) was used to operate the DSC apparatus at the Petroleum and Petrochemical College, Chulalongkorn University. The previous thermal history of the sample was removed before the thermal analysis by heating the sample from ambient temperature to 180°C at 10°C/min. Next, the sample was maintained at 180°C for 5 min before cooling at 10°C/min to −50°C. The sample was then thermally cycled at 10°C/min to 180°C. The melting peak temperature, denoted by Tm, was given by the intersection of the tangent with the furthest point of an endothermic peak and the extrapolated sample baseline. The glass transition temperature, denoted by Tg, could be estimated by extrapolating the midpoint of the heat capacity difference between glassy and viscous states after heating of the quenched sample.

### Data Analysis

All the data presented in this manuscript are representative of the results of three independent experiments and are expressed as the mean values ± standard deviations (SDs). Analysis of variance (one-way ANOVA) followed by Duncan’s test for testing differences among means was conducted using SPSS version 22 (IBM Corp., Armonk, NY, United States). Differences were considered significant at *P* < 0.05.

## Results

### Effect of the Growth Phase on the Production of PhaC_A–04_ and PHB by the Conventional Induction Method

**FIGURE 2:**
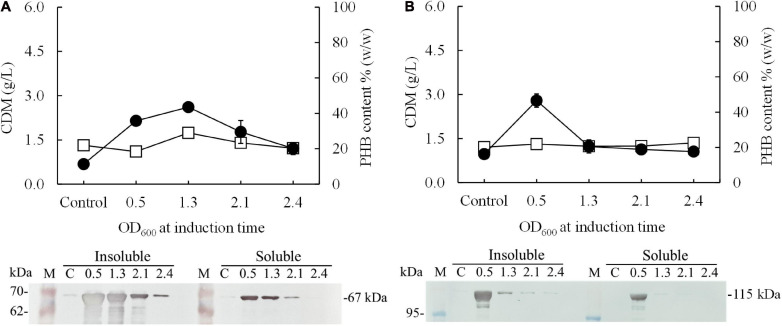
Effect of the growth phase suitable for cold-shock induction on CDM and PHB content (% w/w) under the conventional induction method. The different growth phases were investigated by varying OD_600_ based on cultivation time [0.5 (2 h, early exponential phase), 1.3 (4 h, middle exponential phase), 2.1 (6 h, late exponential phase), and 2.4 (10 h, stationary phase)] for **(A)** *E. coli* JM109 (pColdI-*phaCAB*_A–04_) and **(B)** *E. coli* JM109 (pColdTF-*phaCAB*_A–04_). A control experiment was performed with 0.0 mM IPTG induction. All the data are representative of the results of three independent experiments and are expressed as the mean values ± standard deviations (SDs). The PhaC_A–04_ protein was detected by western blot analysis using anti-His tag antibody as the primary antibody. The band appearing in the western blot at the position corresponding to that of the His-tagged phaC_A–04_ protein was 67 kDa in size for pColdI-*phaCAB*_A–04_, and the fusion protein of His-tagged phaC_A–04_ and TF was 115 kDa in size. All the data are representative of the results of three independent experiments and are expressed as the mean values ± standard deviations (SDs). Symbols: open squares, CDM (g/L); closed circle, PHB (g/L).

In preliminary experiments, after the pColdI-*phaCAB*_A–04_ and pColdTF-*phaCAB*_A–04_ vectors were transformed into *E. coli* JM109, the heterologous expression of *phaCAB*_A–04_ biosynthesis genes was performed by conventional induction method and by varying IPTG concentrations. It was found that 0.5 mM IPTG was the optimal concentration, which was the same as that recommended by the manufacturer’s instructions (Supplementary Figure 2). Next, to optimize the conditions, expression was induced with 0.5 mM IPTG at different growth phases by varying OD_600_ based on cultivation time: 0.5 (2 h, early exponential phase), 1.3 (4 h, middle exponential phase), 2.1 (6 h, late exponential phase), and 2.4 (10 h, stationary phase). Concurrently, the temperature was shifted from 37°C to 15°C for 24 h. Figure 2 shows the effect of the growth phase for gene induction on the CDM (g/L), PHB content (% w/w) and levels of insoluble and soluble PhaC_A–04_ protein, comparing *E. coli* JM109 (pColdI-*phaCAB*_A–04_) and *E. coli* JM109 (pColdTF-*phaCAB*_A–04_). The PhaC_A–04_ protein was detected by western blot analysis using an anti-His tag antibody as the primary antibody. A band appeared in the western blot at the position corresponding to that of the His-tagged phaC_A–04_ protein (67 kDa) for pColdI-*phaCAB*_A–04_ and the fusion protein of His-tagged phaC_A–04_ and TF at 115 kDa. By varying the time courses of the growth phase, His-tagged PhaC_A–04_ and the His-tagged phaC_A–04_-TF fusion protein were successfully expressed, with the highest amount of total phaC_A–04_ protein obtained when the *phaCAB*_A–04_ operon was induced at an OD_600_ of 0.5. The content of soluble PhaC_A–04_-TF fusion protein (Figure 2B, lane 3) in the sample after IPTG induction at an OD_600_ of 0.5 was much higher than that of the phaC_A–04_ protein alone from pColdI-*phaCAB*_A–04_ (Figure 2A, lane 3), suggesting that the TF chaperone facilitates the expression of highly soluble protein in *E. coli* JM109. The highest amount of soluble PhaC_A–04_ and TF fusion protein was produced only at an OD_600_ of 0.5 and was not detected in other growth phases. Functional PhaC_A–04_ protein production was confirmed by determining the amount of PHB produced; however, the value was only 46.2 ± 1.8% w/w with a productivity of 0.03 ± 0.01 g/(L⋅h) (Table 2). Furthermore, the quantification of purified soluble PhaC_A–04_ protein and PhaC_A–04_ and TF fusion protein was performed by IMAC affinity chromatography under native conditions and the results were shown in Table 3 (the details of this experiment are given below).

**TABLE 2:** Effect of IPTG concentration on CDM (g/L), PHB (g/L), % (w/w) PHB content and PHB productivity in a comparison between *E. coli* JM109 harboring pColdI-*phaCAB*_A–04_ and *E. coli* JM109 harboring pColdTF-*phaCAB*_A–04_.

Plasmid	Inoculum % (v/v)	IPTG (mM)	CDM (g/L)	RCM (g/L)	PHB (g/L)	PHB content (% w/w)	Productivity g/(L.h)
**pColdI-*phaCAB*_A–04_**							
Short induction	5	0	2.8 ± 0.1	2.5 ± 0.1	0.3 ± 0.0	10.7 ± 1.1	0.01 ± 0.00
		0.01	2.8 ± 0.2	1.0 ± 0.2	1.8 ± 0.1	64.3 ± 3.1	0.07 ± 0.03
		0.05	2.6 ± 0.3	0.7 ± 0.2	1.9 ± 0.2	73.1 ± 3.5	0.08 ± 0.04
		0.1	2.6 ± 0.2	0.5 ± 0.1	2.1 ± 0.2	80.8 ± 0.7	0.08 ± 0.05
		0.5	4.5 ± 0.3	0.6 ± 0.1	3.9 ± 0.1	86.7 ± 2.6	0.16 ± 0.07
		1	2.6 ± 0.1	0.4 ± 0.0	2.2 ± 0.1	84.6 ± 0.6	0.09 ± 0.02
Conventional induction	5	0.5	1.3 ± 0.1	0.7 ± 0.0	0.6 ± 0.1	46.2 ± 1.8	0.03 ± 0.01
Preinduction	1	0.5	0.8 ± 0.1	0.7 ± 0.2	0.1 ± 0.0	12.5 ± 1.3	0.001 ± 0.00
	5	0.5	2.8 ± 0.6	0.9 ± 0.3	1.9 ± 0.6	67.9 ± 1.8	0.04 ± 0.01
	10	0.5	4.5 ± 1.1	1.0 ± 0.5	3.5 ± 1.1	77.8 ± 2.5	0.07 ± 0.02
**pColdTF-*phaCAB*_A–04_**							
Short induction	5	0	2.5 ± 0.1	2.3 ± 0.2	0.2 ± 0.0	8.0 ± 0.8	0.01 ± 0.00
		0.01	2.5 ± 0.2	1.1 ± 0.2	1.4 ± 0.1	56.0 ± 1.7	0.06 ± 0.02
		0.05	2.7 ± 0.2	0.9 ± 0.1	1.8 ± 0.1	66.7 ± 1.5	0.07 ± 0.03
		0.1	2.8 ± 0.1	0.8 ± 0.2	2.0 ± 0.3	71.4 ± 2.2	0.08 ± 0.08
		0.5	3.5 ± 0.1	0.7 ± 0.2	2.8 ± 0.3	80.0 ± 2.9	0.12 ± 0.07
		1	2.9 ± 0.2	0.7 ± 0.0	2.2 ± 0.2	75.9 ± 0.8	0.09 ± 0.04

*Conventional induction was performed with 0.5 mM IPTG at 15°C, and cultivation was performed at 15°C for 24 h. Short induction was performed with 0.5 mM IPTG at 15°C for 30 min, and cultivation was performed at 37°C for 24 h. Preinduction was performed with 0.5 mM IPTG at 15°C for 24 h, and cultivation was performed at 37°C for 24 h.*

**TABLE 3:** Quantification of purified soluble phaC_A–04_ produced by *E. coli* JM109 (pColdI-*phaCAB*_A–04_) and *E. coli* JM109 (pColdTF-*phaCAB*_A–04_) under short induction method compared with the conventional method in shake flask cultivation.

Strains	Induction method	Initial protein loading (μg)	Total protein obtained after purification (μg)	% recovery	Total soluble his-tagged phaC_A–04_	Soluble his-tagged phaC_A–04_ (%)
pColdI-*phaCAB*_A–04_	Short induction	2,000	1,855 ± 75	93 ± 3.8	66 ± 2.8	3.6 ± 1.4
pColdTF-*phaCAB*_A–04_	Short induction	2,000	1,933 ± 28	97 ± 1.4	287 ± 37	14.8 ± 1.7
pColdI-*phaCAB*_A–04_	Conventional induction	2,000	1,795 ± 53	90 ± 2.7	274 ± 36	15.3 ± 2.3
pColdTF-*phaCAB*_A–04_	Conventional induction	2,000	1,880 ± 106	94 ± 5.3	890 ± 95	47.4 ± 2.4

*The short induction was performed at 15°C for 30 min with 0.5 mM IPTG, and then cultivation was performed at 37°C for 48 h. The conventional induction was performed at 15°C with 0.5 mM IPTG at 15°C, and cultivation was performed at 15°C for 48 h. The experiments were performed as *n* = 3 technical replicates, and the results are expressed as the mean values ± standard errors (SEs).*

### Comparison of the Effect of phaC Expression on PHB Production Under Various Types of Promoters, Fusion Proteins and Chaperones

**FIGURE 3:**
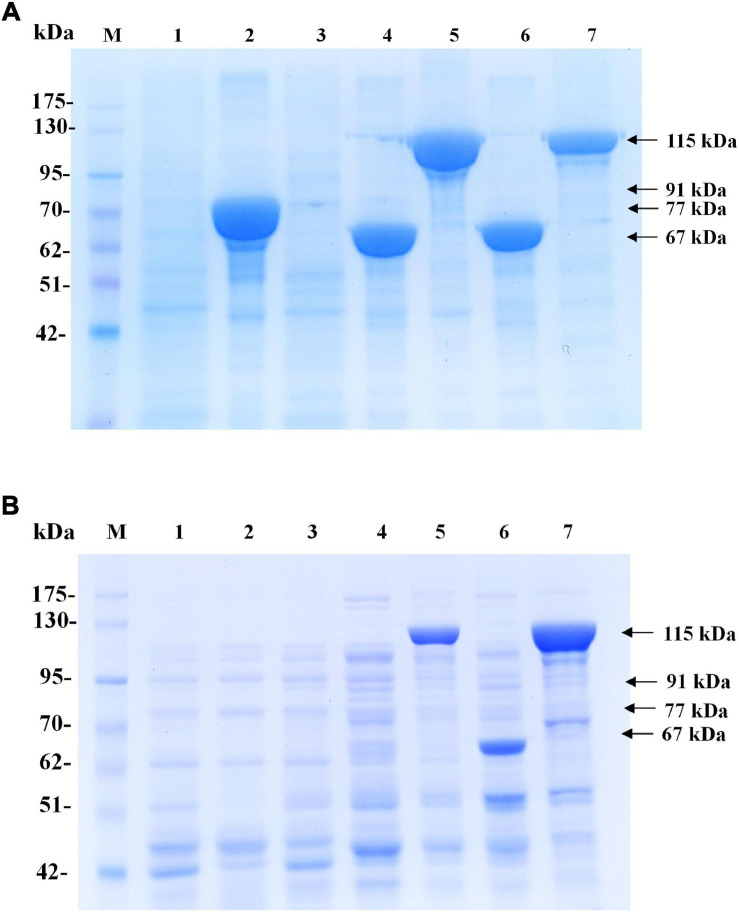
Time courses of PHB production (g/L). **(A)** The insoluble PhaC_A–04_ protein was confirmed by SDS-PAGE analysis (20 μg of total protein was loaded in each lane). **(B)** The soluble PhaC_A–04_ protein was confirmed by SDS-PAGE analysis (20 μg of total protein was loaded in each lane). Lane M, Protein molecular weight marker; lane 1, *E. coli* JM109 (pUC19-nativeP-*phaCAB*_A–04_) under short induction temperature profile, but without addition of IPTG; lane 2, *E. coli* JM109 pBAD/Thio-TOPO- *phaCAB*_A–04_ under short induction method; lane 3, *E. coli* JM109 (pGEX-6P-1- *phaCAB*_A–04_) under short induction method; lane 4, *E. coli* JM109 (pColdI-*phaCAB*_A–04_) under short induction method; lane 5, *E. coli* JM109 (pColdTF-*phaCAB*_A–04_) under short induction method; lane 6, *E. coli* JM109 (pColdI-*phaCAB*_A–04_) under conventional induction method; lane 7, *E. coli* JM109 (pColdTF-*phaCAB*_A–04_) under conventional induction method. The band appearing in the SDS-PAGE at the position corresponding to that of the phaC_A–04_ protein was 64 kDa in size for pUC19-nativeP-*phaCAB*_A–04_, His-tagged phaC_A–04_ fusion protein was 67 kDa in size for pColdI-*phaCAB*_A–04_, thioredoxin-tagged phaC_A–04_ fusion protein was 77 kDa in size for pBAD/Thio-TOPO-*phaCAB*_A–04_, GST-tagged phaC_A–04_ fusion protein was 91 kDa in size for pGEX-6P-1-*phaCAB*_A–04_, and the fusion protein of His-tagged phaC_A–04_ and TF was 115 kDa in size for pColdTF-*phaCAB*_A–04_.

In *phaCAB*_A–04_-overexpressing *E. coli* JM 109 (pColdI-*phaCAB*_A–04_) under the conventional conditions, the formation of inclusion bodies of PhaC_A–04_ has been observed due to the low aqueous solubility of the protein, as described previously (Harada et al., 2019). To verify that the cold-shock cspA promoter works together with the TF chaperone to improve the solubility of PhaC_A–04_, the hydrophilic GST tag was fused to the N-terminus of PhaC_A–04_ (pGEX-6P-1- *phaCAB*_A–04_), and the effect of the GST tag at 37°C on the polymerization reaction of phaC_A–04_ based on the amount of PHB production was investigated. In addition, pBAD/Thio-TOPO- *phaCAB*_A–04_, encoding a hydrophilic N-terminal thioredoxin fusion protein and C-terminal 6His-fusion protein induced by arabinose, was also used for comparison (Napathorn et al., 2021). The control strain, harboring pUC19-nativeP-*phaCAB*_A–04_, was under the control of the native promoter derived from *C. necator* strain A-04, and no induction agent was required under the same conditions. The amounts of PHB are shown in Table 4. The expressed phaC_A–04_ protein was also verified, purified and quantified and shown in Figures 3A,B and Table 3. From Table 4, it was clearly found that pColdI-*phaCAB*_A–04_ and pColdTF-*phaCAB*_A–04_ yielded significantly higher amounts of PHB under the short-induction (15°C for 30 min and then 37°C) conditions than under the conventional induction (15°C) conditions (pColdI-*phaCAB*_A–04_, pColdTF-*phaCAB*_A–04_, pGEX-6P-1-*phaCAB*_A–04_, pBAD/Thio-TOPO-*phaCAB*_A–04_ and pUC19-nativeP-*phaCAB*_A–04_). Next, the expression level of PhaC_A–04_ protein was investigated by SDS-PAGE analysis. Figures 3A,B showed insoluble and soluble PhaC_A–04_ expressed at 24 h of cultivation, respectively. The thioredoxin-tagged PhaC_A–04_ fusion protein (lane 2 at 77 kDa) showed the highest amount of insoluble form, corresponding with a low amount of PHB production. Interestingly, pColdI-*phaCAB*_A–04_ and pColdTF-*phaCAB*_A–04_ produced significantly high amounts of phaC_A–04_ under both the short and conventional induction methods (Figure 3A, lanes 4−7).

**TABLE 4:** Comparison of the kinetics of cell growth, $Y_{\frac{P}{S}}$ (g PHB/g-glucose), and PHB production g/(L.h) by *C. necator* strain A-04, *E. coli* JM109 (pColdI-*phaCAB*_A–04_), (pColdTF-*phaCAB*_A–04_), (pGEX-6P-1- *phaCAB*_A–04_), (pBAD/Thio-TOPO-*phaCAB*_A–04_), and (pUC19-nativeP-*phaCAB*_A–04_) in shake flask cultivation.

Kinetic parameters	Promoters						
	pColdI-*phaCAB*_A–04_	pColdI-*phaCAB*_A–04_	pColdTF-*phaCAB*_A–04_	pColdTF-*phaCAB*_A–04_	pGEX-6P-1- *phaCAB*_A–04_	pBAD/Thio-TOPO- *phaCAB*_A–04_	pUC19-nativeP-*phaCAB*_A–04_
Induction method	A	B	A	B	B	B	B
Maximum PHB concentration (g/L)	1.4 ± 0.2	2.6 ± 0.2	1.3 ± 0.1	2.5 ± 0.1	0.9 ± 0.2	0.8 ± 0.2	0.7 ± 0.1
Maximum CDM (g/L)	1.7 ± 0.1	2.9 ± 0.2	1.7 ± 0.2	2.8 ± 0.1	1.3 ± 0.1	1.2 ± 0.2	1.1 ± 0.2
Maximum PHB content (%w/w)	82.4 ± 2.5	89.7 ± 0.8	76.5 ± 3.3	89.3 ± 4.3	69.2 ± 2.6	66.7 ± 1.8	63.6 ± 2.2
Specific growth rate (1/h)	0.001	0.001	0.001	0.001	0.003	0.003	0.004
Specific consumption rate (g glucose/g CDM/h)	1.03	0.75	0.73	1.13	0.56	0.31	0.5
Specific production rate (g PHB/g CDM/h)	0.09	0.19	0.07	0.29	0.05	0.03	0.05
*Y*_*X / S*_ (g CDM/g glucose)	0.001	0.002	0.01	0.001	0.008	0.026	0.01
*Y*_*P / a*_ (g PHB/g glucose)	0.07	0.18	0.08	0.18	0.08	0.10	0.06
Productivity (g/(L.h)	0.03	0.09	0.03	0.10	0.02	0.03	0.02
Time (h)	48	30	48	24	48	30	30

***A:** The conventional induction was performed with 0.5 mM IPTG at 15°C, and cultivation was performed at 15°C for 48 h. **B:** The short induction was performed with 0.5 mM IPTG at 15°C for 30 min, and then, cultivation was performed at 37°C for 48 h.*

To clarify and carefully compare the amounts of soluble phaC_A–04_ expressed by pColdI-*phaCAB*_A–04_ and pColdTF-*phaCAB*_A–04_ under short induction and conventional induction, the phaC_A–04_ protein was purified by IMAC affinity chromatography under native conditions. The eluted fractions were quantified by Bradford protein assay. The results are summarized in Table 3. The figures of SDS-PAGE and Western blot analysis were represented as Supplementary Results. The initial protein loading was adjusted to 2,000 μg and the maximum capacity of the IMAC column was 3,000 μg. The protein recovery was within the range of 90–97%. The conventional method induced soluble phaC_A–04_ from pColdTF-*phaCAB*_A–04_ at a level of as high as 47.4% of total protein and pColdTF-*phaCAB*_A–04_ enhanced soluble protein formation to approximately 3.09−4.1 times higher than that from pColdI-*phaCAB*_A–04_ by both conventional method and short induction method. Based on our observations, the cold-shock cspA promoter enhanced phaC_A–04_ protein expression and TF promoted soluble phaC_A–04_ protein (Figure 3B and Table 3). The PHB production from pGEX-6P-1-*phaCAB*_A–04_, pBAD/Thio-TOPO-*phaCAB*_A–04_ and pUC19-nativeP-*phaCAB*_A–04_ was not different, which may be attributed to the host strain and induction method used in this study. Therefore, pColdTF-*phaCAB*_A–04_ and pColdI-*phaCAB*_A–04_ were chosen to validate their effectiveness of PHB production in the 5 L fermenter.

### Development of Short-Induction Method and Pre-induction Method

**FIGURE 4:**
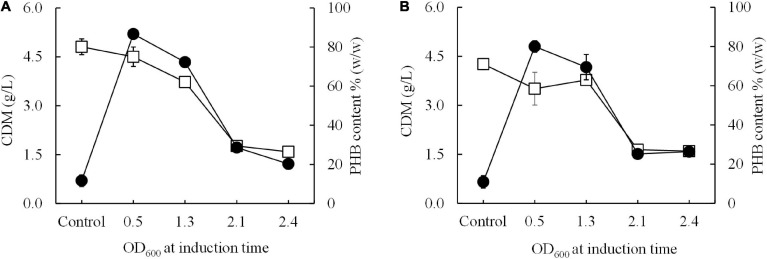
Effect of the growth phase suitable for cold-shock induction on CDM and PHB content (% w/w) under the short-induction method. The different growth phases were investigated by varying OD_600_ based on cultivation time [0.5 (2 h, early exponential phase), 1.3 (4 h, middle exponential phase), 2.1 (6 h, late exponential phase), and 2.4 (10 h, stationary phase)] for **(A)** *E. coli* JM109 (pColdI-*phaCAB*_A–04_) and **(B)** *E. coli* JM109 (pColdTF-*phaCAB*_A–04_). A control experiment was performed with 0.0 mM IPTG induction. All the data are representative of the results of three independent experiments and are expressed as the mean values ± standard deviations (SDs). Symbols: open squares, CDM (g/L); closed circle, PHB (g/L).

Next, a short-induction method was investigated in this study with the aim of accelerating growth and PHB production and attaining higher productivity than that afforded by the conventional induction method. First, conditions were optimized by varying the OD_600_ based on cultivation time (0.5, 1.3, 2.1, and 2.4 h) and inducing expression with 0.5 mM IPTG at 15°C for 30 min. Then, the temperature was shifted from 15°C to 37°C for 24 h to enhance growth and PHB production. The effect of the growth phase (OD_600_) on CDM (g/L) and PHB content (% w/w) is illustrated in Figure 4. Again, it was clearly observed that cells of both *E. coli* JM109 (pColdI-*phaCAB*_A–04_) and *E. coli* JM109 (pColdTF-*phaCAB*_A–04_) in the 2-h early exponential phase (OD_600_ of 0.5) exhibited higher CDM and PHB production than those in other growth phases. After induction with 0.5 mM IPTG at 15°C for 30 min and cultivation at 37°C for 24 h, *E. coli* JM109 (pColdI-*phaCAB*_A–04_) attained 4.5 ± 0.3 g/L CDM, 3.9 ± 0.1 g/L PHB and 86.70 ± 2.6% (w/w) PHB content with a productivity of 0.16 ± 0.07 g PHB/(L.h), whereas *E. coli* JM109 (pColdTF-*phaCAB*_A–04_) attained 3.5 ± 0.1 g/L CDM, 2.8 ± 0.3 g/L PHB and 80.0 ± 2.9% (w/w) PHB content with a productivity of 0.12 ± 0.07g PHB/(L.h). Thus, the short-induction method enhanced the PHB content and productivity more than the conventional method. Next, an OD_600_ of 0.5 was used to investigate the optimal concentration of IPTG (0, 0.01, 0.05, 0.1, 0.5, and 1.0 mM) under the short-induction conditions. The effects of various IPTG concentrations on CDM (g/L), PHB (g/L), PHB content (% w/w) and PHB productivity [g PHB/(L.h)], comparing *E. coli* JM109 (pColdI-*phaCAB*_A–04_) and *E. coli* JM109 (pColdTF-*phaCAB*_A–04_), are summarized in Table 2. It can be concluded that the optimal concentration of IPTG was 0.5 mM in both cases. The PHB content (% w/w) increased in accordance with the IPTG concentration, but the amount of PHB (g/L) produced was maximum under induction with 0.5 mM IPTG. The PHB content (% w/w) increased approximately 8-fold, and the productivity [g PHB/(L.h)] increased 16-fold, compared with those under the control condition in the case of pColdI-*phaCAB*_A–04_.

**FIGURE 5:**
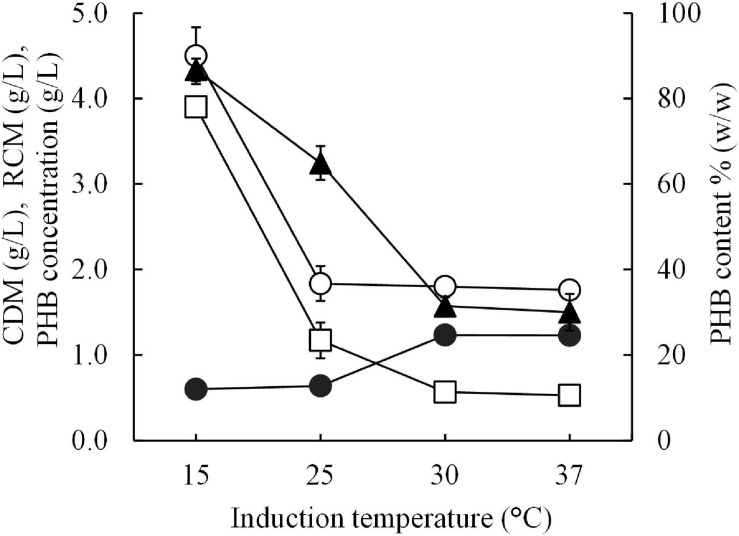
Effect of different short-induction temperatures (15, 25, 30, and 37 °C) on the CDM (g/L), RCM (g/L), PHB (g/L), and PHB content (% w/w) of *E. coli* JM109 (pColdI-*phaCAB*_A–04_). All the data are representative of the results of three independent experiments and are expressed as the mean values ± standard deviations (SDs). Symbols: open circle, CDM (g/L); closed circle, RCM (g/L); open square, PHB (g/L); closed triangle, PHB content (% w/w).

The optimal short-induction temperature was investigated in a range between 15°C and 37°C for 30 min before increasing the temperature to 37°C for 24 h to confirm that the high PHB productivity resulting in this study is a result of the cold-shock cspA promoter and that 15°C is the optimal induction temperature. Figure 5 shows the results of the effect of the short-induction temperature (15, 25, 30, and 37°C) on cell growth and PHB production. It was clear that 15°C was the optimal induction temperature for enhancing the amount of PHB produced, which resulted in a maximum PHB content of 86.7 ± 2.6% (w/w). The amount of PHB produced decreased as the induction temperature increased. The PHB productivity at 15°C was sevenfold higher than that obtained with an induction temperature of 37°C.

We also investigated a preinduction strategy to enhance PHB productivity by extending the PHB production phase at 37°C for an additional 24 h after conventional induction. When the OD_600_ reached 0.5, IPTG was added at 0.5 mM into the culture, and the temperature was reduced from 37 to 15°C. Then, cultivation was performed for 24 h to allow full expression of the phaCAB_A–04_ protein. Concurrently, the effect of inoculum size [1, 5, and 10% (v/v)] of induced cells was investigated under the preinduction conditions. The results are shown in comparison with those of the conventional induction and short-induction methods (Table 2). The preinduction method with a 5% (v/v) inoculum gave a higher amount of PHB (1.9 ± 0.6 g/L) than conventional induction with an inoculum size of 5% (v/v) (0.6 ± 0.1 g/L) and could extend the productivity of 0.039 ± 0.01 g PHB/(L⋅h) for 48 h so that the PHB content increased from 46.2 ± 1.8% (w/w) to 67.9 ± 1.8% (w/w). The increase in PHB content and PHB productivity occurred with an increase in the inoculum size. Nevertheless, the short-induction method with an inoculum size of 5% (v/v) gave the highest levels of PHB content and productivity. Therefore, the short-induction method using *E. coli* JM109 (pColdI-*phaCAB*_A–04_) with an inoculum size of 0.5% (v/v) and cultivated until the OD_600_ reached 0.5 (2 h) before induction with 0.5 mM IPTG was selected to investigate the effect of induction temperature in the subsequent experiment.

### Comparison of PHB Production Between pColdI-*phaCAB*_A–04_ and pColdTF-*phaCAB*_A–04_ in a 5-L Fermentor by the Short Induction Method

Altogether, for flask cultivation, the optimal conditions were the short-induction method using an inoculum of 0.5% (v/v) in a culture with an OD_600_ of 0.5, cold shock induced with 15°C for a short time, 30 min, and the addition of 0.5 mM IPTG. These conditions were selected as optimal parameters for scaling up production in a 5-L fermenter. The comparison between pColdI-*phaCAB*_A–04_ and pColdTF-*phaCAB*_A–04_ in a 5-L fermenter by the short-induction method was performed because the ratio of the soluble fraction and inclusion bodies of the phaC_A–04_ protein may affect PHB productivity and molecular weight distribution as reported by Harada et al. (2019).

**FIGURE 6:**
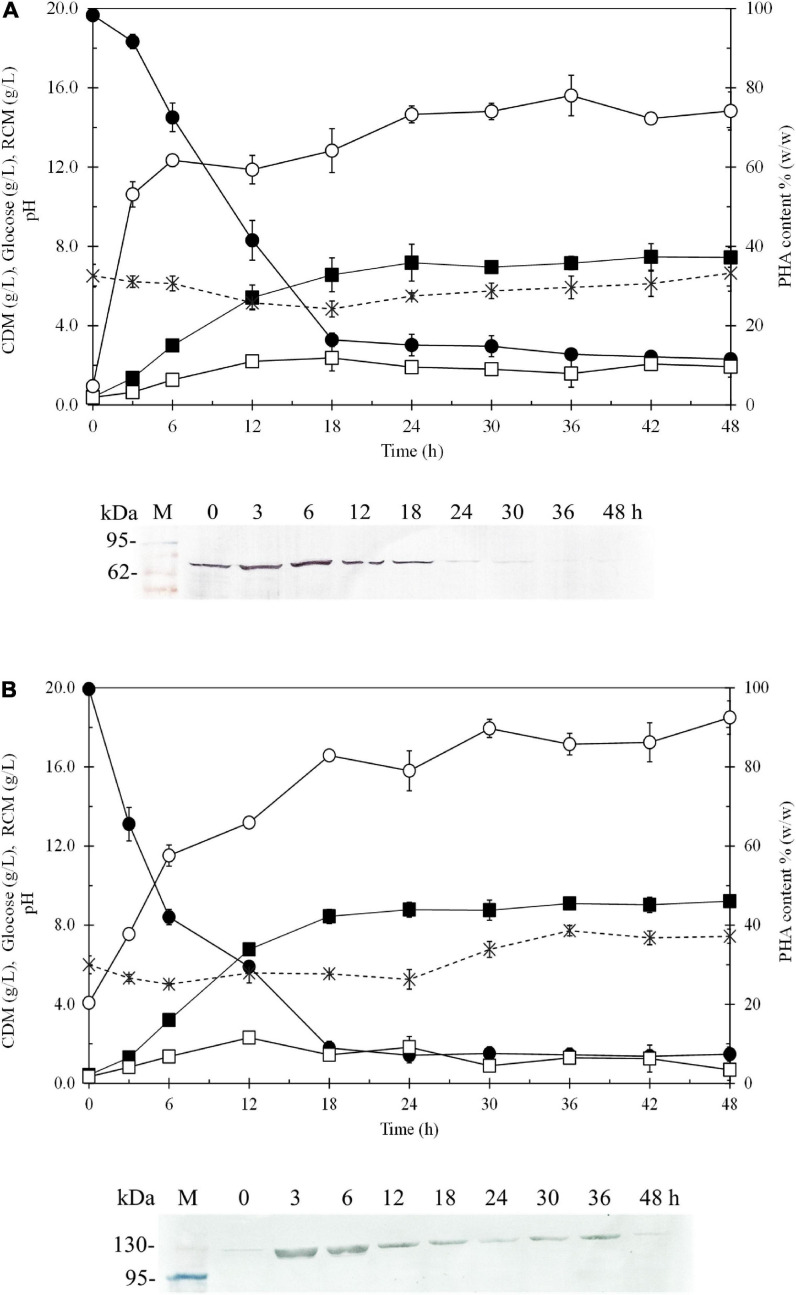
Time courses of CDM (g/L), RCM (g/L), PHB (g/L), PHB content (% w/w), and glucose (g/L) and pH during batch cultivation in a 5-L fermenter under the short-induction method in a comparison between **(A)** *E. coli* JM109 (pColdI-*phaCAB*_A–04_) and **(B)** *E. coli* JM109 (pColdTF-*phaCAB*_A–04_). The band appearing in the western blot at the position corresponding to that of the His-tagged phaC_A–04_ protein was 67 kDa in size for pColdI-*phaCAB*_A–04_, and the fusion protein of His-tagged phaC_A–04_ and TF was 115 kDa in size. All the data are representative of the results of three independent experiments and are expressed as the mean values ± standard deviations (SDs). Symbols: closed square, CDM (g/L); closed circle, glucose (g/L); asterisks, pH; open square, RCM (g/L); open circle, PHB content (% w/w).

Figure 6 shows the time courses of CDM (g/L), RCM (g/L), PHB (g/L), PHB content (% w/w), glucose (g/L), dissolved oxygen (%) and pH during batch cultivation in a 5-L fermenter, comparing *E. coli* JM109 (pColdI-*phaCAB*_A–04_) (Figure 6A) and *E. coli* JM109 (pColdTF-*phaCAB*_A–04_) (Figure 6B). The soluble PhaC_A–04_ protein detected by western blot analysis was also monitored at 6-h intervals over 48 h. The results shown in Table 5 demonstrated that *E. coli* JM109 (pColdTF-*phaCAB*_A–04_) was a more effective PHB producer than the other strain. A PHB content of 89.8 ± 2.3% (w/w), PHB production of 7.9 ± 0.7 g/L, CDM production of 8.8 ± 0.5 g/L, Y_P/S_ value of 0.38 g PHB/g glucose and productivity of 0.43 g PHB/(L.h) were the maximum values obtained using pColdTF-*phaCAB*_A–04_, whereas a PHB content of 80.6 ± 2.1% (w/w), PHB production of 5.8 ± 0.1 g/L, CDM production of 7.2 ± 0.3 g/L, Y_P/S_ value of 0.32 g PHB/g glucose and productivity of 0.24 g PHB/(L.h) were attained using pColdI-*phaCAB*_A–04_. The phaC_A–04_ protein produced by pColdTF-*phaCAB*_A–04_ was more stable and longer lasting (Figure 6B) than that obtained from pColdI-*phaCAB*_A–04_, which was no longer detectable after 30 h of cultivation (Figure 6A). Therefore, we report here that the short-induction strategy facilitates cold shock cspA and chaperone TF proteins to act synergistically to improve the stabilization of PhaC_A–04_ and enhance productivity and the Y_P/S_ value in comparison with the use of cspA alone in pColdI.

**TABLE 5:** Comparison of kinetic parameters, molecular weight, and thermal and mechanical properties of PHB produced by *C. necator* strain A-04, *E. coli* JM109 (pColdI-*phaCAB*_A–04_), *E. coli* JM109 (pColdTF-*phaCAB*_A–04_), and *E. coli* JM109 (pUC19-nativeP-*phaCAB*_A–04_) in 5-L fermenter.

Kinetic parameters and polymer properties of PHB	*C. necator* A-04		pColdTF-*phaCAB*_A–04_	pColdI- *phaCAB*_A–04_		pUC19-nativeP- *phaCAB*_A–04_
	Fructose	Raw sugar	Glucose	Glucose	Crude glycerol	Crude glycerol
Carbon source (g/L)	20	30	20	20	20	20
Maximum PHB concentration (g/L)	5.8 ± 0.5	4.7 ± 0.2	7.9 ± 0.7	5.8 ± 0.1	2.0 ± 0.1	2.1 ± 0.1
Maximum cell dry weight (g/L)	7.4 ± 1.5	7.3 ± 1.2	8.8 ± 0.5	7.2 ± 0.3	4.0 ± 0.2	3.9 ± 0.3
Maximum PHB content (%wt)	78.4 ± 1.9	64.4 ± 2.8	89.8 ± 2.3	80.6 ± 2.1	50.0 ± 3.0	53.8 ± 2.2
Specific growth rate (1/h)	0.003	0.001	0.07	0.06	0.08	0.11
Specific consumption rate (g carbon source/g CDM/h)	0.14	0.05	0.52	0.35	0.20	0.19
Specific production rate (g PHB/g CDM/h)	0.012	0.019	0.20	0.11	0.02	0.016
*Y*_*X*/*S*_ (g CDM/g-carbon source)	0.08	0.03	0.07	0.10	0.19	0.16
*Y*_*P*/*S*_ (g PHB/g carbon source)	0.29	0.35	0.38	0.32	0.13	0.09
Productivity [g/(L.h)]	0.10	0.07	0.43	0.24	0.11	0.07
M_w_ (×10^5^)	6.51	3.30	5.79	8.41	2.42	10.68
M_n_ (×10^5^)	3.61	1.46	1.86	2.03	0.89	2.60
PDI	1.80	2.15	3.11	4.14	2.92	4.10
Young’s modulus (MPa)	1497	1734	5465	2156	1980	2262
Tensile strength (MPa)	17.4	11.9	56.2	21.5	19.3	17.4
Elongation at break (%)	0.4	1.1	1.2	1.7	2.0	1.1
T_m_ (°C)	178	173	168	176	170	174
T_g_ (°C)	2.4	3.5	1.6	3.0	1.9	2.8
Time (h)	60	60	18	30	18	30

**FIGURE 7:**
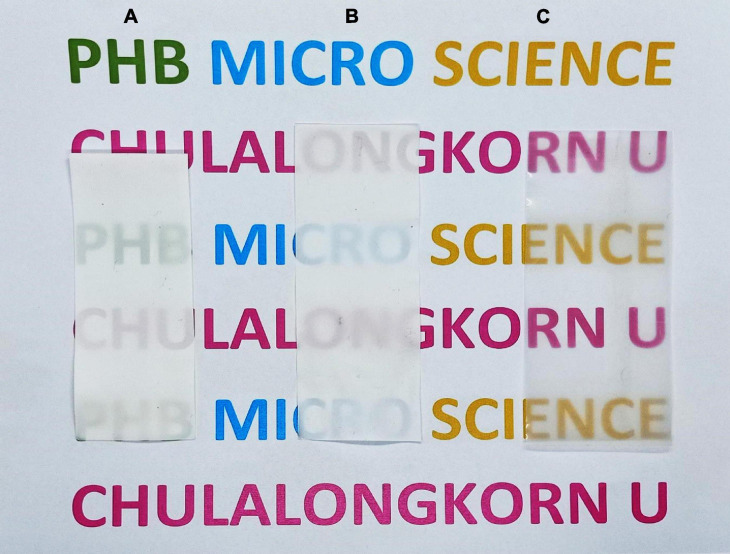
Morphology of PHB films produced by **(A)** *C. necator* strain A-04, **(B)** *E. coli* JM109 (pColdI-*phaCAB*_A–04_), and **(C)** *E. coli* JM109 (pColdTF-*phaCAB*_A–04_).

The PHB thin films were subjected to thermal analysis by DSC, molecular weight determination by GPC and mechanical property analysis by a universal testing machine as per the ASTM: D882-91 protocol (Table 5). The PHB from *E. coli* JM109 (pColdI-*phaCAB*_A–04_) had an M_w_ of 8.17 × 10^5^ Da, an M_n_ of 1.97 × 10^5^ Da and a PDI of 4.1, whereas the PHB from E. coli JM109 (pColdTF-*phaCAB*_A–04_) had an M_w_ of 2.6 × 10^5^ Da, an M_n_ of 0.95 × 10^5^ Da and a PDI 2.8, when glucose was used as a carbon source. However, the PHB from *E. coli* JM109 (pColdI-*phaCAB*_A–04_) obtained using crude glycerol had the lowest M_w_ of 2.42 × 10^5^ Da, an M_n_ of 0.89 × 10^5^ Da and a PDI of 2.92. Interestingly, the PHB from *E. coli* JM109 (pUC19-nativeP-*phaCAB*_A–04_) obtained using crude glycerol showed the highest M_w_ of 1.1 × 10^6^ Da, an M_n_ of 2.6 × 10^5^ Da and a PDI of 4.1. The melting temperature, T_m_, of all the PHB film samples produced in this study was in the range of 165−178°C (Owen et al., 1992), and the glass transition temperature, T_g_, was in the normal range of 1−4°C (Shimamura et al., 1994; Doi et al., 1995; Chanprateep et al., 2010). The Young’s modulus and tensile strength of the PHB from *E. coli* JM109 (pColdTF-*phaCAB*_A–04_) possessed the highest values of 5465 and 56.2 MPa, respectively. Figure 7 shows the morphology and transparency of PHB films produced by *C. necator* strain A-04, *E. coli* JM109 (pColdI-*phaCAB*_A–04_) and *E. coli* JM109 (pColdTF-*phaCAB*_A–04_). The PHB films prepared by the film casting technique and produced from *E. coli* JM109 (pColdTF-*phaCAB*_A–04_) showed a soft morphology with high transparency, which was different from the properties of the other PHB films. The PHB films were also subjected to ^1^H-NMR and ^13^C-NMR analyses and showed only chemical shifts of the PHB structure.

## Discussion

It has been reported that PhaC_H16_, as a type I synthase and the most intensively studied of these proteins, preferentially catalyzes the polymerization of short-chain (R)-hydroxyalkanoic acids (4 to 6 carbon atoms), particularly the conversion of (R)-3-hydroxybutyrate-coenzyme A (3HBCoA) to poly(3-hydroxybutyrate) (PHB) (Schubert et al., 1988; Gerngross et al., 1994). In fact, a high concentration of PHB (157 g/L) has been achieved from glucose in high-cell-density cultures of recombinant *E. coli* harboring *phaCAB*_H16_ and the additional cell division protein ftsZ gene (Wang and Lee, 1997; Choi and Lee, 2004). Ultrahigh-molecular-weight PHB and its applications have also been reported by many research groups (Kusaka et al., 1998; Iwata, 2005; Hiroe et al., 2012; Kabe et al., 2012). Beyond these previous reports, there have been few reports on the application of cold-shock systems for PHB production to address the challenges of soluble phaC expression in *E. coli*. From our previous reports, *C. necator* strain A-04 exhibits 99.78% similarity of 16S rRNA, 99.9% similarity of *phaC*_A–04_ and 100% similarity of *phaA*_A–04_ and *phaB*_A–04_ with those of *C. necator* H16. However, we observed differences in PHB productivity as well as the monomeric composition of the copolymers and terpolymers when we used the same carbon source (Chanprateep and Kulpreecha, 2006; Chanprateep et al., 2008, 2010). Interestingly, *C. necator* strain A-04 also exhibited growth abilities on pure glycerol as well as crude glycerol from a biodiesel plant in Thailand as compared with *C. necator* strain H16 (unpublished observations from personal communication with Tuck Seng Wong, University of Sheffield, United Kingdom). Thus, *C. necator* strain A-04 can be considered as one of the promising candidates for PHA production. However, the PHA productivity of *C. necator* strain A-04 still does not meet the requirement for industrial production. Thus, our objective was to investigate the ability of the phaCAB_A–04_ gene operon when heterologously expressed in recombinant *E. coli*. We initially aimed to use the pColdI and pColdTF expression systems to address the challenges of soluble and functional phaC_A–04_ expression in *E. coli* JM109 from glucose as a carbon source for PHB production in a 5-L fermenter and evaluate its ability to use crude glycerol to attain low cost PHB production.

First, this study aimed to increase soluble PhaC_A–04_ expression in *E. coli* JM109 by using the cold-shock cspA promoter and TF. His-tagged phaC_A–04_ was overexpressed by pColdI, but most of the protein was present in insoluble form, with significant aggregation resulting in smear bands (Figures 2A, 5 and Table 3), whereas the His-tagged phaC_A–04_-TF fusion protein was expressed from pColdTF at lower levels than the protein from pColdI, but most of this protein was present in soluble form.

To examine the optimal induction temperature, the cold shock temperature was varied at 15, 25, 30, and 37°C for 30 min and then the cultivation temperature was shifted to 37°C for 24 h. It is noted that the optimal growth temperature of *E. coli* is 37°C where the optimal growth temperature for *C. necator* strain A-04 is 30°C. Our results demonstrated that the PHB content decreased when the temperature increased although the optimal growth temperature for *C. necator* strain A-04 was 30°C. The appropriate temperature for enzymatic activity of phaC_A–04_ activity can be considered between 30 to 37°C similar to Sheu et al. (2012) who reported the effects of temperature on the activity of PHA synthases and who revealed that mesophilic (PhaC_H16_) PHA synthases were differentiated distinctly based on the highest level of PHB accumulation at different temperatures. They demonstrated that the parental enzymes PhaC_H16_ had optimal temperature of 37°C (Sheu et al., 2012). Thus, our strategy (short induction) for PHB production using a cold-shock promoter in *E. coli* is lowering the temperature at 15°C for 30 min and then shifting the temperature to 37°C which is the optimal temperature for phaC activity.

We also performed parallel experiments using different hydrophilic tags, including expression via the native promoter of *C. necator* strain A-04, N-terminal GST-fused *phaCAB*_A–04_, and N-terminal thioredoxin-fused and C-terminal 6His-fused *phaCAB*_A–04_, to confirm that the high efficiency of PHB production was contributed by the cold-shock cspA promoter. It was found that the GST-PhaCAB_A–04_, Thioredoxin-PhaCAB_A–04_, 6His-phaCAB_A–04_ and nativeP-pha*CAB*_A–04_ gave similar values for PHB production and PHB content, which were 2.5 times lower than the values obtained with pCold and pColdTF (Figure 3A and Table 4). In our study, the GST-PhaC_A–04_ did not improve PHB production and exhibited lower PHB productivity than the control pUC19-nativeP-*phaCAB*_A–04_, same as previously mentioned by Harada et al. (2019). The *araBAD* promoter showed the highest level of PhaC_A–04_ protein production, but most of them were in insoluble form. However, this study was performed using the short induction strategies that could limit the expression of PhaCAB_A–04_ under different promoters.

In addition, the ratio of soluble fraction to the total phaC_A–04_ proteins from pColdTF-*phaCAB*_A–04_ and pColdI-*phaCAB*_A–04_ under conventional induction and short induction method was carefully analyzed by IMAC affinity chromatography under native conditions protein (Table 3). The ratio of soluble fraction to the total phaC_A–04_ proteins from pColdTF-*phaCAB*_A–04_ was about 3−4 times higher than that from pColdI-*phaCAB*_A–04_ both under short induction and conventional induction method. Thus, it can be concluded that the TF chaperone helped solubilize phaC_A–04_ in our investigation. Finally, the production of PHB of pColdI-*phaCAB*_A–04_ and pColdTF-*phaCAB*_A–04_ was compared in the 5-L fermenter using the short induction method and yielded Y_P/S_ of 0.38 g-PHB/g-glucose whereas the theoretical yield Y_P/S_ was reported as 0.48 of *g*-PHB/*g*-glucose (Yamane, 1993). This phenomenon can be explained that the growth of *E. coli* on glucose causes acidic by-products formation, mainly acetate, under both aerobic and anaerobic conditions (Lee et al., 1994). In this study, it was observed that the pH decreased from 7.0 (initial pH) to 4.9–5.0 at 18 h as acetate accumulated and then increased back to 6.6–7.4 (Figure 6) as acetate was consumed (Chen et al., 2018). Therefore, the Y_P/S_ obtained in these experiments was lower than the theoretical yield. In this study, the pH and dissolved oxygen were not controlled because Lee et al. reported that controlled pH and/or dissolved oxygen of the recombinant *E. coli* strains resulted in higher RCM with low PHB content of less than 40% due to better growth condition, where much acetyl-CoA can flow into the tricarboxylic acid (TCA) cycle (Lee et al., 1994). Acetyl-CoA is a fundamental metabolite in central metabolic pathways of *E. coli*, and also served as a precursor for biosynthesis of a large number of industrial chemicals and natural products including PHB (Martin et al., 2003; Meng et al., 2012; Liu et al., 2016; Sun et al., 2020). The main metabolic route for acetyl-CoA synthesis in *E. coli* is the glycolysis pathway coupled with decarboxylation of pyruvate by pyruvate dehydrogenase (Bates et al., 1977). Through this pathway, each mol of glucose is converted into 2 mol of acetyl-CoA with generation of 4 mol of NADH, 2 mol of ATP and 2 mol of CO_2_. The release of CO_2_ lowers the atomic economy of targeted chemical biosynthetic pathway, leading to the decrease of theoretical production yield, titer and productivity (Chae et al., 2017). In addition, glucose is catabolized through glycolysis pathway to pyruvate, which is converted into acetyl-CoA under catalysis of pyruvate dehydrogenase, with reduction of NAD+ to NADH in both glycolysis pathway and pyruvate dehydrogenation. As a result, NADH could not be oxidized through respiratory chain sufficiently, the build-up of NADH rapidly inactivated the pyruvate dehydrogenase (Hansen and Henning, 1966), leading to accumulation of pyruvate in cells, and the recycling of NAD+ must be achieved through the reduction of some metabolites (Bunch et al., 1997). Therefore, acetate is accumulated in medium in large quantity. Acetate overflow is caused by an imbalance between the pathways of glycolysis and TCA cycle in rapidly growing cells, and severely decreases the yield of target chemicals from glucose (Farmer and Liao, 1997; Wong et al., 2008). It has been known that *E. coli* cells grown on glucose produce acetate and consume acetate after glucose exhaustion, but do not grow on acetate due to the decoupling of acetate anabolism and acetate catabolism, and the growth restores only after prolonged exposure to acetate (Sun et al., 2020). With the same glucose concentration, Lee et al. (1994) performed a comparative study of recombinant *E. coli* strains (K12, B, W, XL1-Blue, JM109, DH5α, and HB101) for PHB production from glucose. They reported that Y_P/S_ of *E. coli* strain XL1-Blue (pSYL105) grown in LB medium containing 20 g/L glucose was as high as 0.369 g PHB/g glucose whereas JM109 provided 0.299 g PHB/g glucose. Thus, the Y_P/S_ obtained in this study was comparable. In the fed-batch cultivation studies, Wang and Lee (1997) performed a fed-batch culture of *E. coli* XL1-Blue (pSYL107) in a 50-l fermentor and attained Y_P/S_ of 0.28 g of PHB/g of glucose (3,849 g of PHB produced from 13,900 g of glucose). They mentioned that a slightly lower PHB yield on glucose in a defined medium than in LB medium (0.37 g of PHB/g of glucose) because glucose was converted to CO_2_ in a defined medium more than in LB medium (Wang and Lee, 1997). Next, they applied the feeding solution contained 700 g/L glucose, 20 MgSO_4_⋅7H_2_O, and 250 mg/L of thiamine in fed-batch cultivation. As a result, cell concentration of 149 g/L and PHB concentration of 104 g/L (PHB content 69.5%) were obtained in 51 h, resulting in the PHB productivity of 2.0 g/L/h. This is the highest PHB concentration obtained by employing recombinant *E. coli* containing the *phaCAB*_H16_ biosynthesis genes and the *E. coli* ftsZ gene. In our previous report, we performed fed-batch cultivations by pH-stat control in a 5-L fermenter using *E. coli* strain XL1-Blue harboring pColdTF-*phaCAB*_A–04_, leading to a PHB production of 31.4 ± 0.9 g/L at 54 h with a PHB content of 83.0 ± 3.8% (w/w), a CDM of 37.8 ± 1.2 g/L, a Y_P/S_ value of 0.39 g PHB/g glucose and a productivity of 0.6 g PHB/(L⋅h) in define medium. To investigate the possibility of improving the PHB yield and productivity, we are now carrying out fed-batch cultures under various conditions including oxygen limitation during PHB synthesis phase as it may be possible to enhance the PHB synthesis rate by increasing the acetyl-CoA flux into the PHB biosynthetic pathway and reducing its flux into the tricarboxylic acid cycle (Wang and Lee, 1997).

The produced PHB were characterized and it was found that the M_w_ of PHB produced from pColdTF-*phaCAB*_A–04_, for which soluble phaC_A–04_ was 4.1 times higher than pColdI-*phaCAB*_A–04_ was lower than that from pColdI-*phaCAB*_A–04_. Hiroe et al. (2012) have reported that that the concentration of active PHA synthase had a negative correlation with PHB molecular weight and a positive correlation with cellular PHB content, similar to our observation. The M_w_ and M_n_ of PHB produced by pColdTF were lower than those of pColdI-*phaCAB*_A–04_ and pUC19-nativeP-pha*CAB*_A–04_. In this case, TF increases PhaC production and its Mw decreases due to the presence of more active PhaC. To achieve low-cost production, crude glycerol containing 80% glycerol provided by Bangchak Corporation Public Company Limited as a byproduct from biodiesel production, was used as a carbon source to produce PHB using pColdI-*phaCAB*_A–04_ and pUC19-nativeP-pha*CAB*_A–04_. With *E. coli* JM109 (pColdI-*phaCAB*_A–04_), PHB produced from crude glycerol had an M_w_ of 2.42 × 10^5^ Da and an M_n_ of 0.89 × 10^5^ Da with a PDI of 2.92, whereas those results from glucose were M_w_ of 8.41 × 10^5^ Da and an M_n_ of 2.03 × 10^5^ Da with a PDI of 4.14. The crude glycerol caused the termination step involves a chain transfer (CT) reaction in which the polymer chain is transferred to the crude glycerol (CT agent) in this case (Madden et al., 1999). However, in our study, when pUC19-nativeP-*phaCAB*_A–04_-expressing *E. coli* was used to produce PHB from crude glycerol, an M_w_ of 1.1 × 10^6^ Da, an M_n_ of 2.6 × 10^5^ Da and a PDI of 4.1 were obtained, indicating that slow and low phaC_A–04_ expression prolonged and maintained the phaC_A–04_ polymerization activity, which in turn resulted in a low amount of PHB with a high molecular weight (Hiroe et al., 2012).

## Conclusion

This study revealed that the cspA promoter in a cold-inducible vector can enhance total PhaC_A–04_ expression and TF chaperones showed obvious effects on enhancing PhaC solubility. The short induction strategies developed in this study did not affect on molecular weight distribution and polymer properties. Cultivation in a 5-L fermenter led to PHB production of 7.9 ± 0.7 g/L with 89.8 ± 2.3% PHB content in the cell dry mass (CDM), a Y_P/S_ value of 0.38 g PHB/g glucose and a productivity of 0.43 g PHB/(L⋅h) using pColdTF-*phaCAB*_A–04_. The PHB from pColdTF-*phaCAB*_A–04_ had Mw 5.79 × 10^5^ Da, Mn 1.86 × 10^5^ Da and PDI 3.11 and the film exhibited high optical transparency with typical melting temperature and mechanical properties.

## Data Availability Statement

The datasets presented in this study can be found in online repositories. The names of the repository/repositories and accession number(s) can be found below: https://www.ncbi.nlm.nih.gov/genbank/, FJ897463; https://www.ncbi.nlm.nih.gov/genbank/, FJ897461; and https://www.ncbi.nlm.nih.gov/genbank/, FJ897462.

## Author Contributions

TB performed the experiments and discussed the results. RW-S provided guidance for the experimental design and discussed the results. KH provided suggestions for the experimental design and discussed the results. SCN provided guidance and suggestions for the experimental design, discussed the results, and wrote and revised the manuscript. All the authors read and approved the final version of the manuscript.

## Conflict of Interest

The authors declare that the research was conducted in the absence of any commercial or financial relationships that could be construed as a potential conflict of interest.

## Supplementary Material

Supplementary Figure 1 Construction of PHA biosynthesis genes in cold-shock inducible expression vectors **(A)** pColdI-*phaCAB*_A–04_ and **(B)** pColdTF-*phaCAB*_A–04_.

Supplementary Figure 2 Effect of IPTG concentrations (0.01, 0.05, 0.1, 0.5, and 1.0 mM) on the expression of His-tagged phaC_A–04_ protein **(A)** (pColdI-*phaCAB*_A–04_) and **(B)** the fusion protein of His-tagged phaC_A–04_ and TF (pColdTF-*phaCAB*_A–04_) under conventional induction method. The band appearing in the SDS PAGE at the position corresponding to that of the His-tagged phaC_A–04_ protein was 67 kDa in size and the fusion protein of His-tagged phaC_A–04_ and TF was 115 kDa in size.

Supplementary Figure 3 Purification of His-tagged PhaC_A–04_ protein of *E. coli* JM109 (pColdI-*phaCAB*_A–04_) at 15°C **(A)** and the fusion protein of His-tagged PhaC_A–04_ protein and TF of *E. coli* JM109 (pColdTF-*phaCAB*_A–04_) at 15°C by IMAC affinity chromatography under native condition **(B)**. The extracted protein was normalized to 2 mg and loaded on Protino^®^ Ni-IDA 1000 packed column. Ten microliters of each fraction eluted from IMAC column were loaded onto 10% w/v acrylamide gel for SDS-PAGE and Western blot analysis. M, Protein molecular weight marker; Ly, Bacterial lysate, soluble proteins; Fl, Flow-though lysate; W1 and W2, Wash with 50 mM NaH_2_PO_4_, 300 mM NaCl, 20 mM imidazole and pH 8.0; E1–E3, Eluted with 50 mM NaH_2_PO_4_, 300 mM NaCl, 250 mM imidazole and pH 8.0. His-tagged phaC_A–04_ fusion protein was 67 kDa in size for pColdI-*phaCAB*_A–04_ and the fusion protein of His-tagged phaC_A–04_ and TF was 115 kDa in size for pColdTF-*phaCAB*_A–04_. The soluble fractions were quantified by Bradford protein assay. All IMAC purifications were performed as *n* = 3 technical replicates.
