# Supplementary figures and images for: Strategies for Poly(3-hydroxybutyrate) Production Using a Cold-Shock Promoter in Escherichia coli
Source: Front Bioeng Biotechnol. 2021 Jun 3;9:666036. doi: 10.3389/fbioe.2021.666036 (PMC8211017; doi:10.3389/fbioe.2021.666036)

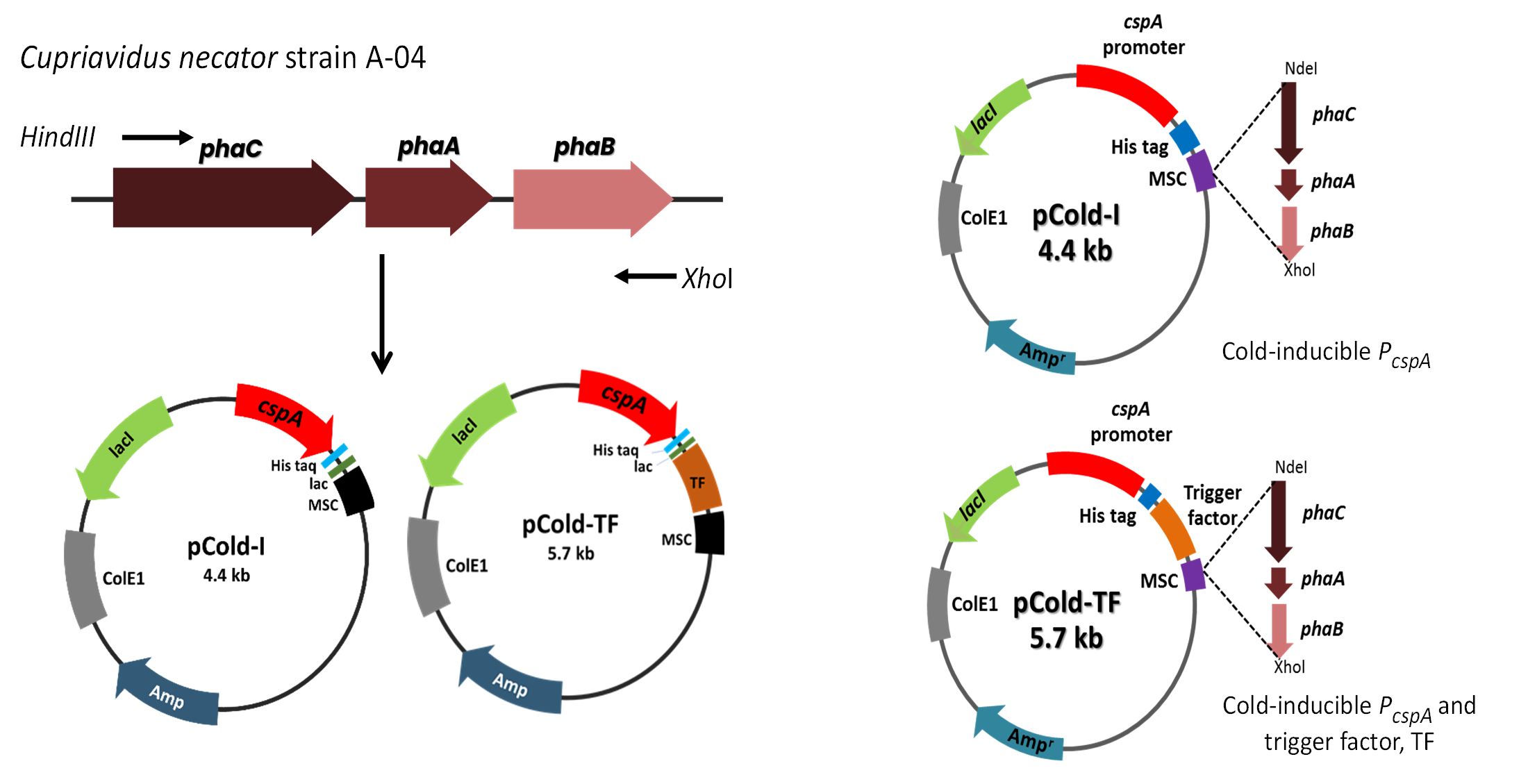

Supplement: Supplementary Figure 1 — Construction of PHA biosynthesis genes in cold-shock inducible expression vectors (A) pColdI-phaCABA–04 and (B) pColdTF-phaCABA–04. [file Image_1.tif]

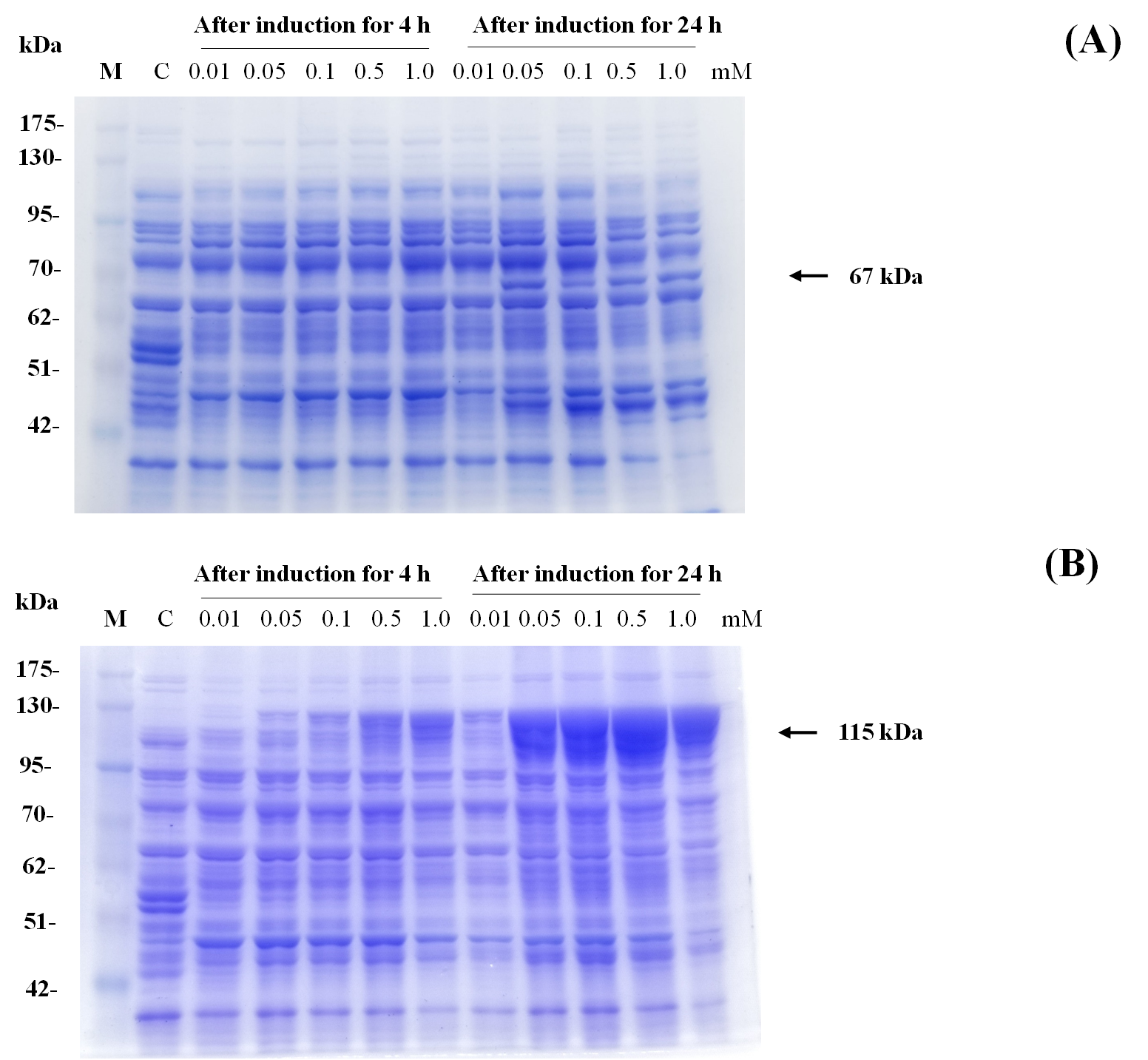

Supplement: Supplementary Figure 2 — Effect of IPTG concentrations (0.01, 0.05, 0.1, 0.5, and 1.0 mM) on the expression of His-tagged phaCA–04 protein (A) (pColdI-phaCABA–04) and (B) the fusion protein of His-tagged phaCA–04 and TF (pColdTF-phaCABA–04) under conventional induction method. The band appearing in the SDS PAGE at the position corresponding to that of the His-tagged phaCA–04 protein was 67 kDa in size and the fusion protein of His-tagged phaCA–04 and TF was 115 kDa in size. [file Image_2.tif]

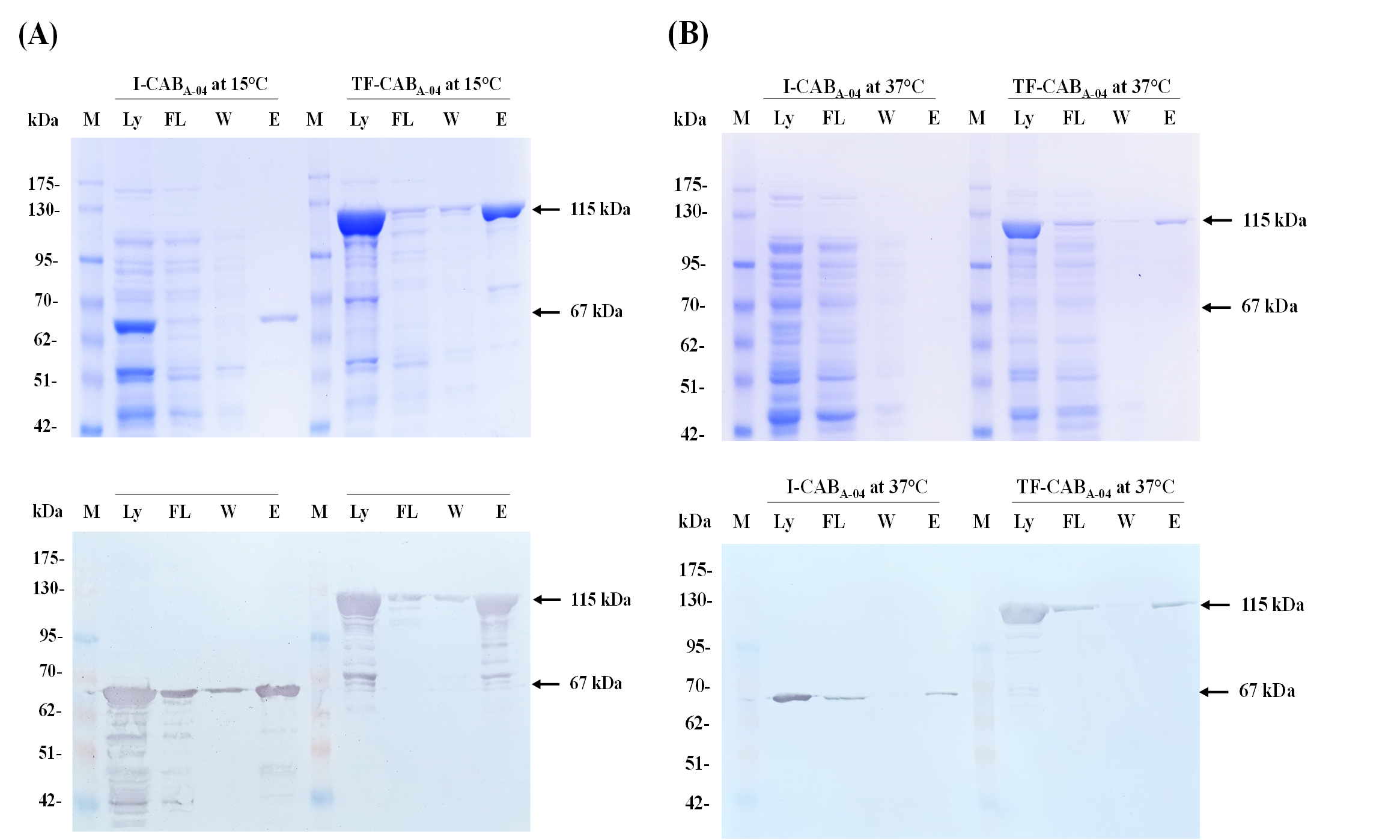

Supplement: Supplementary Figure 3 — Purification of His-tagged PhaCA–04 protein of E. coli JM109 (pColdI-phaCABA–04) at 15°C (A) and the fusion protein of His-tagged PhaCA–04 protein and TF of E. coli JM109 (pColdTF-phaCABA–04) at 15°C by IMAC affinity chromatography under native condition (B). The extracted protein was normalized to 2 mg and loaded on Protino® Ni-IDA 1000 packed column. Ten microliters of each fraction eluted from IMAC column were loaded onto 10% w/v acrylamide gel for SDS-PAGE and Western blot analysis. M, Protein molecular weight marker; Ly, Bacterial lysate, soluble proteins; Fl, Flow-though lysate; W1 and W2, Wash with 50 mM NaH2PO4, 300 mM NaCl, 20 mM imidazole and pH 8.0; E1–E3, Eluted with 50 mM NaH2PO4, 300 mM NaCl, 250 mM imidazole and pH 8.0. His-tagged phaCA–04 fusion protein was 67 kDa in size for pColdI-phaCABA–04 and the fusion protein of His-tagged phaCA–04 and TF was 115 kDa in size for pColdTF-phaCABA–04. The soluble fractions were quantified by Bradford protein assay. All IMAC purifications were performed as n = 3 technical replicates. [file Image_3.tif]
